# Beyond Maximum Haemorrhage Grade: Serial Cranial Ultrasound Phenotyping Refines Developmental Risk Stratification After Intraventricular Haemorrhage in Very Preterm Infants

**DOI:** 10.3390/jcm15166310

**Published:** 2026-08-14

**Authors:** Noemí Núñez-Enamorado, Ana Camacho-Salas, María López-Maestro, María Carmen Gallego-Herrero, Sara Vila-Bedmar, Sara Vázquez-Román, Berta Zamora-Crespo, Noelia Ureta Velasco, Elisa Aguirre Pascual, Carmen Rosa Pallás-Alonso, María Teresa Moral-Pumarega

**Affiliations:** 1 Division of Paediatric Neurology, Hospital Universitario 12 de Octubre, Avenida de Córdoba s/n, 28041 Madrid, Spain; acamacho@salud.madrid.org (A.C.-S.); sara.vila@salud.madrid.org (S.V.-B.); 2 Faculty of Medicine, Complutense University of Madrid, 28040 Madrid, Spain; carmenrosa.pallas@salud.madrid.org (C.R.P.-A.); mmoralp@salud.madrid.org (M.T.M.-P.); 3 Neonatology Unit, Hospital Universitario 12 de Octubre, Avenida de Córdoba s/n, 28041 Madrid, Spain; mlmaestro@salud.madrid.org (M.L.-M.); sara.vazquez@salud.madrid.org (S.V.-R.); noelia.ureta@salud.madrid.org (N.U.V.); 4 Division of Paediatric Radiology, Hospital Universitario 12 de Octubre, Avenida de Córdoba s/n, 28041 Madrid, Spain; cgallego@salud.madrid.org (M.C.G.-H.); elisa.aguirre@salud.madrid.org (E.A.P.); 5 Division of Paediatric Neuropsychology, Hospital Universitario 12 de Octubre, Avenida de Córdoba s/n, 28041 Madrid, Spain; berta.zamora@salud.madrid.org

**Keywords:** neonatal intensive care, very preterm infant, intraventricular haemorrhage, cranial ultrasound, ventriculomegaly, post-haemorrhagic hydrocephalus, white-matter injury, cerebral palsy, neurodevelopmental outcome, risk stratification

## Abstract

**Background/Objectives**: In neonatal intensive care, intraventricular haemorrhage (IVH) is one of the most frequent brain morbidities in very preterm infants, but maximum IVH grade may not fully capture developmental risk. We assessed whether a serial cranial ultrasound (CUS) phenotype integrating IVH grade, associated parenchymal lesion visible on serial CUS (visible APL) and ventricular morbidity refined developmental risk stratification. **Methods**: We studied 2756 very preterm infants from a single-centre cohort of 3081 who had at least two protocolised CUS examinations. IVH was classified by maximum serial CUS grade. Visible APL, severe ventriculomegaly (VM), post-haemorrhagic hydrocephalus (PHH), cerebral palsy (CP), clinically classified school-age cognitive sequelae and sustained developmental/educational support were analysed. **Results**: Visible APL and ventricular morbidity increased with maximum IVH grade. Among infants with grade 3 IVH (IVH3), visible APL was present in 60.5%, severe VM in 40.8% and PHH in 21.7%. In the isolated-IVH analysis, CP occurred in 1.4% of the reference group (no IVH/no visible APL), 2.9% with isolated grade 1 IVH, 2.8% with isolated grade 2 IVH and 27.5% with IVH3. Isolated IVH3 was associated with CP (aOR 28.55, 95% CI 11.31–72.11), school-age cognitive sequelae and sustained support. After adding severe VM, the CP aOR for isolated IVH3 attenuated to 12.94, while severe VM remained associated with CP, cognitive sequelae and support. **Conclusions**: Our findings support interpreting IVH as a multidimensional serial CUS risk-stratification phenotype beyond maximum grade alone, with the potential to inform bedside counselling and targeted developmental follow-up in neonatal intensive care, pending external validation.

## 1. Introduction

Very preterm infants are at substantial risk of neonatal morbidity, brain injury and subsequent neurodevelopmental impairment [1,2,3,4,5,6]. Among neonatal morbidities, germinal matrix–intraventricular haemorrhage (IVH) is one of the most common cranial ultrasound (CUS) abnormalities and remains a key marker for neurodevelopmental risk stratification, counselling and follow-up planning [7,8,9,10,11].

Maximum IVH grade is strongly associated with adverse neurodevelopmental outcomes. Meta-analyses and population-based cohorts have demonstrated a graded increase in cerebral palsy (CP), cognitive impairment and neurodevelopmental disability with increasing IVH severity [12,13,14,15,16,17,18,19,20,21,22]. These data confirm that maximum IVH grade provides an important first layer of risk stratification. However, the same maximum grade may encompass clinically distinct serial CUS phenotypes. Historical grading systems included periventricular haemorrhagic infarction (PVHI) within “grade 4 IVH”, whereas contemporary classification recognises PVHI as a parenchymal venous infarction with distinct pathophysiology and prognosis [8,9,15]. Contemporary reviews also emphasise that outcome after IVH may be influenced by coexisting white-matter injury, PVHI, ventricular dilatation and secondary effects on brain maturation [8,9,10,23]. A further source of heterogeneity is the neurodevelopmental significance of low-grade IVH: some studies and meta-analyses have reported increased risks after grade 1–2 IVH, whereas others have found modest or no clear association when co-occurring parenchymal brain injury is absent [12,13,16,17,19,20,21]. One explanation is that “isolated IVH” has not been defined uniformly across studies, and associated parenchymal lesions visible on neonatal neuroimaging may not always have been separated from haemorrhage grade itself [8,20,21].

Post-haemorrhagic ventricular dilatation is another clinically relevant component of neurodevelopmental risk stratification after IVH. Progressive ventricular dilatation and hydrocephalus, including post-haemorrhagic hydrocephalus (PHH) after IVH, have been associated with adverse motor and cognitive outcomes, altered brain maturation and clinically relevant treatment thresholds [24,25,26,27,28,29,30,31]. However, ventricular dilatation is often analysed as a complication of high-grade IVH or mixed haemorrhagic–parenchymal injury, rather than as a feature that may refine developmental risk when IVH occurs without visible parenchymal lesion. This distinction is particularly relevant for grade 3 IVH, where infants assigned the same maximum IVH grade may still differ substantially in their serial ventricular trajectory, from stable ventricular size to severe ventriculomegaly or clinically documented hydrocephalus/PHH.

Serial CUS provides a practical bedside framework to determine whether IVH is accompanied by visible parenchymal lesion, document the maximum grade and its evolution, and characterise the ventricular course during neonatal admission [7,8,9,11,32]. Previous studies have addressed important components of this problem by separating IVH from white-matter or parenchymal injury, examining low-grade IVH, or confirming graded associations between IVH severity and adverse outcomes in meta-analyses [13,16,17,19,20,21]. However, haemorrhage grade, visible parenchymal lesion status and ventricular course have rarely been integrated within a single serial CUS-based framework linked to developmental outcomes.

In this study, we used a large single-centre cohort of very preterm infants (<32 weeks’ gestation and/or birth weight (BW) ≤ 1500 g) with protocolised serial CUS during neonatal intensive-care admission to evaluate IVH as a developmental risk-stratification phenotype. We first described how maximum IVH grade related to associated parenchymal lesion visible on serial CUS (visible APL) and to ventricular morbidity, including severe ventriculomegaly and PHH. We then focused on IVH without visible APL (isolated IVH) to compare developmental outcomes across isolated IVH grades 1, 2 and 3. We further assessed whether severe ventricular morbidity refined risk stratification beyond maximum IVH grade. We hypothesised that low-grade isolated IVH would show low absolute CP risk, whereas isolated grade 3 IVH would remain a clinically meaningful risk phenotype, with risk further stratified by severe ventriculomegaly and hydrocephalus/PHH. Secondary analyses using the full CUS phenotype and refined visible parenchymal-lesion categories were used to contextualise the contribution and heterogeneity of visible APL.

## 2. Materials and Methods

### 2.1. Study Design, Setting and Cohort

This retrospective single-centre cohort study was conducted in the tertiary neonatal intensive-care setting of Hospital Universitario 12 de Octubre, Madrid, Spain. Eligible infants were live-born very preterm infants, defined as <32 weeks’ gestation and/or BW ≤ 1500 g, delivered at the study centre or admitted within the first 48 h of life between 1991 and 2020. The source cohort comprised 3081 infants. The main serial CUS cohort included infants with at least two complete CUS examinations. Birth epoch was categorised as 1991–2000, 2001–2010 and 2011–2020 and included as a prespecified covariate. Additional details on cohort assembly, birth epochs, exclusions and survival/imaging conditioning are provided in Supplementary Method S1.

### 2.2. Serial CUS Protocol, Window Selection and Data Abstraction

Serial CUS was performed as part of routine neonatal intensive-care imaging using a protocolised four-window approach: within the first 48 h after birth, around day 7, around day 28, and near term-equivalent age or discharge, whichever occurred first. CUS examinations were performed and reported in routine clinical practice by the paediatric radiology team, including senior paediatric radiologists. At each selected window, IVH grade, ventriculomegaly (VM) grade and parenchymal lesion pattern were recorded. A CUS window was considered complete when all three domains were available. Details of within-window scan selection, completeness criteria and missing imaging data are provided in Supplementary Method S2. CUS findings were extracted from contemporaneous routine clinical ultrasound reports recorded in the neonatal registry. Systematic blinded re-review of stored images was not performed, interobserver agreement was not assessed, and MRI was not systematically available across birth epochs or used to define the primary imaging phenotypes. Changes in ultrasound technology, acquisition practice and reporting detail across the study period are addressed in Supplementary Method S3.

### 2.3. Imaging Definitions and CUS Phenotype Classification

IVH was classified as absent or graded from 1 to 3 according to the Papile classification as modified by Volpe [33,34]. PVHI was analysed separately as an APL rather than as an additional IVH grade. In bilateral cases, the highest grade recorded on either side was used. Maximum IVH grade was defined as the highest grade recorded across available serial CUS windows.

Parenchymal lesion pattern was graded using an institutional 0–4 ultrasound scale adapted from de Vries and colleagues [35], with level 0 indicating absence of visible APL. Visible APL was analysed as a study-specific registry-derived composite variable encompassing parenchymal abnormalities recorded on routine serial CUS, including PVHI, cystic white-matter injury, non-cystic periventricular echodensities, focal haemorrhagic or ischaemic lesions, deep grey-matter or cerebellar lesions, multicystic encephalomalacia and other major focal/structural abnormalities. The visible APL composite was used primarily to determine whether IVH occurred without any parenchymal abnormality visible on serial CUS; it was not intended to imply equivalent biological or prognostic significance across its component lesion types. Isolated IVH was defined as IVH grade 1–3 without visible APL. VM and hydrocephalus/PHH were not used to define isolated IVH and were analysed separately as ventricular morbidity variables. This definition does not exclude subtle parenchymal, cerebellar or microstructural abnormalities detectable only by MRI. Detailed definitions and visible APL subtypes are provided in Supplementary Method S4.

VM was graded at each CUS window using a registry-based institutional 0–3 ultrasound scale based on standard neonatal ventricular assessment. Maximum VM grade was defined as the highest grade recorded across available serial CUS windows. Severe VM, defined as maximum VM grade 3, was selected as the main ventricular trajectory variable; moderate-to-severe VM, defined as maximum VM grade ≥ 2, was examined in sensitivity analyses. Severe VM and hydrocephalus/PHH were treated as hierarchically related but analytically distinct variables. Severe VM was treated as the imaging-severity variable, whereas hydrocephalus was treated as a clinicoradiological clinical-course variable defined by progressive ventricular dilatation on serial CUS accompanied by clinical signs suggestive of raised intracranial pressure and/or the need for temporising or definitive cerebrospinal-fluid diversion. In infants with IVH, hydrocephalus was considered post-haemorrhagic hydrocephalus (PHH). Non-dilative intracranial hypertension was not classified as PHH. All infants with PHH also met criteria for severe VM, whereas severe VM could occur without clinically documented PHH. PHH was therefore analysed as an exploratory clinical subset representing the most severe ventricular course. VM grading rules, measurement thresholds and detailed definitions of hydrocephalus/PHH are provided in Supplementary Method S5.

For phenotype analyses, infants were classified into six mutually exclusive CUS phenotype groups: no IVH/no visible APL; no IVH with visible APL; isolated grade 1 IVH (IVH1); isolated grade 2 IVH (IVH2); isolated grade 3 IVH (IVH3); and IVH with visible APL. The full CUS phenotype framework is detailed in Supplementary Method S6.

### 2.4. Clinical Variables and Developmental Outcomes

Baseline variables included GA, BW, sex, multiple gestation, 5 min Apgar score, birth epoch and survival status before discharge. CP was the primary clinical outcome and was analysed as absent or present. Among children with CP, clinical phenotype and Gross Motor Function Classification System-equivalent severity grade were summarised descriptively. Secondary developmental outcomes included clinically classified school-age cognitive sequelae and sustained developmental/educational support. Clinically classified school-age cognitive sequelae were derived from available longitudinal clinical, neurodevelopmental and educational follow-up records and were not treated as a harmonised psychometric endpoint. Sustained developmental/educational support was analysed as a pragmatic functional outcome and included maintained support starting before 2 years, maintained preschool support or support first recorded at primary-school age. Transient early support only was not classified as sustained support. Follow-up pathways, outcome definitions, availability and missingness patterns are described in Supplementary Method S7.

### 2.5. Statistical Analysis

Continuous variables are presented as median and interquartile range, and categorical variables as *n*/*N* (%). Descriptive group comparisons used non-parametric or categorical tests, as appropriate; *p* values in descriptive tables were interpreted as indicators of group differences. Primary adjusted analyses were performed within the isolated-IVH analysis framework, using infants without IVH and without visible APL as the reference group. Model A included isolated IVH grade group, categorised as isolated IVH1–2 and isolated IVH3, and was adjusted for GA, sex and birth epoch. Model B additionally included severe VM to assess whether ventricular trajectory refined developmental risk stratification beyond maximum IVH grade; this was not interpreted as a formal mediation analysis. The adjustment set was intentionally parsimonious and focused on core baseline and temporal factors. BW was not included in the primary models because of collinearity with GA, but sensitivity models replacing GA with BW were used. Postnatal morbidities and treatments were not included in the primary models because they may occur after the imaging exposure, may lie on the clinical pathway between prematurity, brain injury and outcome, and were not uniformly suited to causal adjustment across the three-decade cohort. Separate multivariable logistic regression models were fitted for CP, clinically classified school-age cognitive sequelae and sustained developmental/educational support. Results are presented as adjusted odds ratios with 95% confidence intervals. Analyses were performed on an available-case basis without imputation. Secondary and sensitivity analyses are detailed in Supplementary Method S8 and included full CUS phenotype models, VM ≥ 2 models, exploratory hydrocephalus/PHH models, sustained support analyses among children without CP and/or clinically classified cognitive sequelae, penalised logistic regression for sparse CP models, and birth-weight-adjusted sensitivity models. Analyses were designed to characterise associations within a CUS-defined clinical framework and refine developmental risk stratification, not to support causal inference or to develop or validate a prediction model. Main analyses were performed using Python version 3.13.5 with pandas version 2.2.3 and statsmodels version 0.14.6.

### 2.6. Generative Artificial Intelligence Use

During the preparation of this manuscript, ChatGPT (OpenAI, San Francisco, CA, USA) was used for wording refinement and to assist in the generation of graphical material. The authors reviewed and edited all AI-assisted output and take full responsibility for the content of the manuscript. No generative artificial intelligence tool was used to analyse the study dataset, generate study results or interpret the data.

## 3. Results

### 3.1. Analytic Cohort

**Figure 1:**
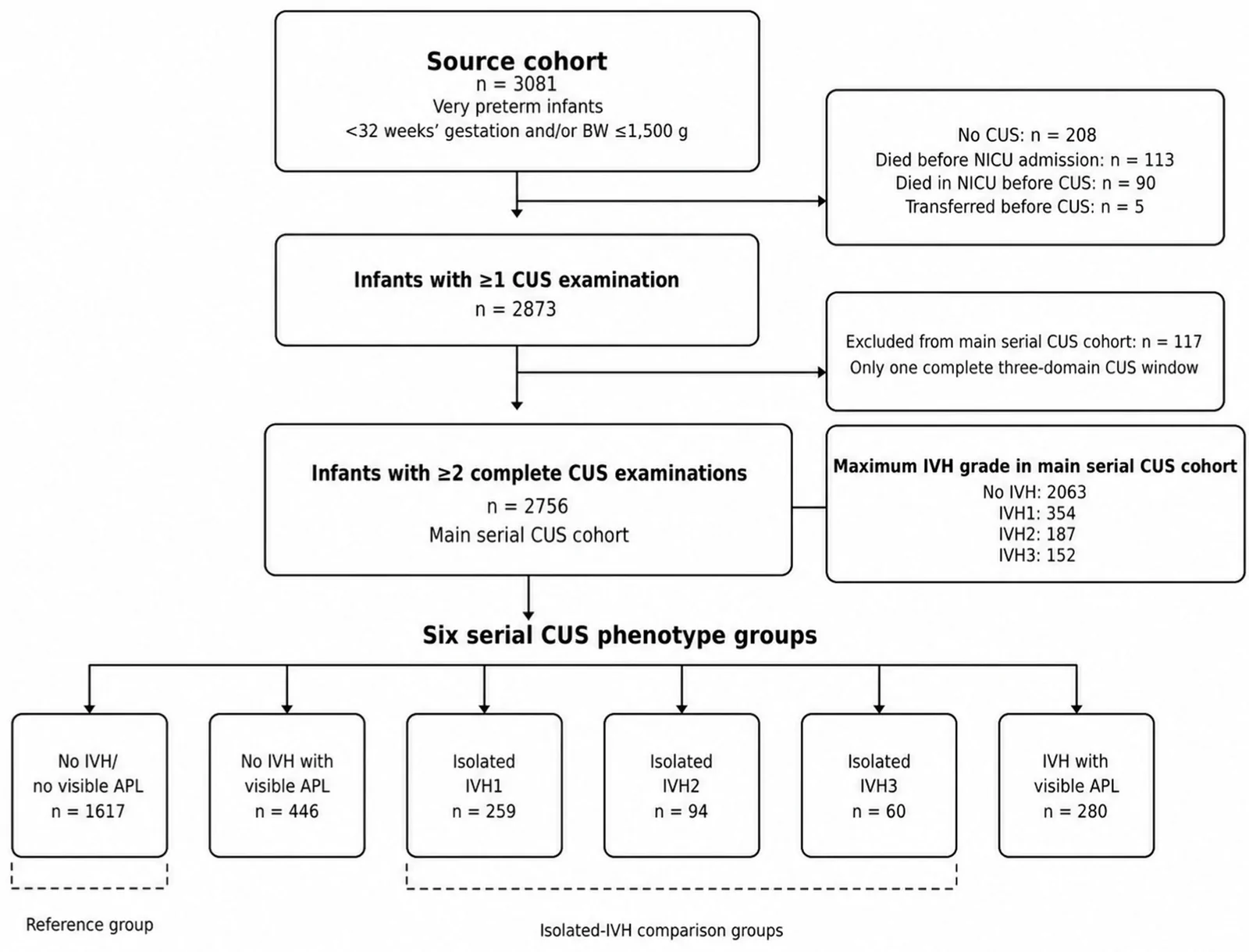
Study flow diagram and serial CUS phenotype classification. Figure 1 shows the derivation of the main serial CUS cohort and the six mutually exclusive serial CUS phenotype groups. The main serial CUS cohort included infants with at least two complete CUS examinations. A complete CUS window required available IVH grade, VM grade and parenchymal lesion pattern. Visible APL was a broad CUS-defined composite used primarily to determine whether IVH was isolated and did not imply equivalent prognostic significance across lesion subtypes. Its composition and secondary subtype-specific analyses are reported in Supplementary Tables S9 and S12. Isolated IVH was defined as IVH grade 1–3 without visible APL. The dashed brackets identify the reference group (no IVH/no visible APL) and the isolated-IVH comparison groups (isolated IVH1–3) used in the main developmental analyses. Detailed CUS window availability, reasons for exclusion and comparison of included and excluded infants are provided in Supplementary Tables S1 and S2. Abbreviations: APL, associated parenchymal lesion; BW, birth weight; CUS, cranial ultrasound; IVH, intraventricular haemorrhage; NICU, neonatal intensive-care unit; VM, ventriculomegaly.

Of 3081 infants in the source cohort, 2756 (89.5%) had at least two complete CUS examinations and formed the main serial CUS cohort (Figure 1; Supplementary Table S1). Availability of at least two complete CUS examinations increased across birth epochs, from 717/856 (83.8%) in 1991–2000 to 1207/1292 (93.4%) in 2011–2020 (Supplementary Table S1). Infants excluded from the main serial CUS cohort were substantially more immature and had higher mortality than included infants, reflecting predominantly early deaths before completion of serial imaging (median GA 25 vs. 29 weeks; median BW 660 vs. 1150 g; death before discharge 97.2% vs. 7.1%; Supplementary Table S2). Complete availability across all four CUS windows was lower in infants with more severe imaging phenotypes, particularly those with IVH3 or IVH with visible APL (Supplementary Table S1). Accordingly, developmental outcome analyses were conditional on survival, completion of sufficient serial imaging and follow-up availability.

### 3.2. Baseline Characteristics, CUS Phenotype Distribution and Timing of IVH Evolution

The main serial CUS cohort was distributed across the six mutually exclusive CUS phenotype groups shown in Figure 1 and Table 1. Higher-severity IVH phenotypes, particularly isolated IVH2, isolated IVH3 and IVH with visible APL, were characterised by lower GA, lower BW, lower 5 min Apgar scores and lower survival to discharge than the no IVH/no visible APL group (Table 1). Survival to discharge declined from 96.2% in infants without IVH/no visible APL to 81.7% in isolated IVH3 and 78.6% in IVH with visible APL. Follow-up availability varied by outcome and CUS phenotype, particularly for clinically classified school-age cognitive outcomes, and is detailed in Supplementary Tables S3 and S4.

Maximum IVH grade was inversely related to GA: IVH2–3 decreased from 34.6% at <26 weeks to 3.2% at ≥30 weeks, and IVH3 from 13.0% to 1.3% (Supplementary Table S5). Across birth epochs, any IVH remained broadly stable, whereas IVH3 was lower in 2011–2020. GA and birth epoch were therefore retained as prespecified adjustment variables in the primary models. The timing and evolution of IVH differed by maximum grade. Grade 2 IVH was usually detected at the maximum grade on the first CUS showing haemorrhage (155/187, 82.9%), whereas grade 3 IVH more often evolved from a lower grade detected on an earlier CUS (80/152, 52.6%) (Supplementary Tables S6 and S7).

### 3.3. Associated Parenchymal and Ventricular Morbidity Across Maximum IVH Grade Categories

Higher maximum IVH grade was accompanied by a progressively greater frequency of co-occurring visible parenchymal lesions and ventricular morbidity (Table 2). The proportion of infants with visible APL increased from 26.8% in IVH1 to 49.7% in IVH2 and 60.5% in IVH3; conversely, only 39.5% of infants with IVH3 had no visible APL. Ventricular dilatation was also concentrated in IVH3: severe VM was uncommon in IVH1 and IVH2 but was present in 40.8% of IVH3 cases, and PHH occurred in 21.7% of IVH3 cases.

**Figure 2:**
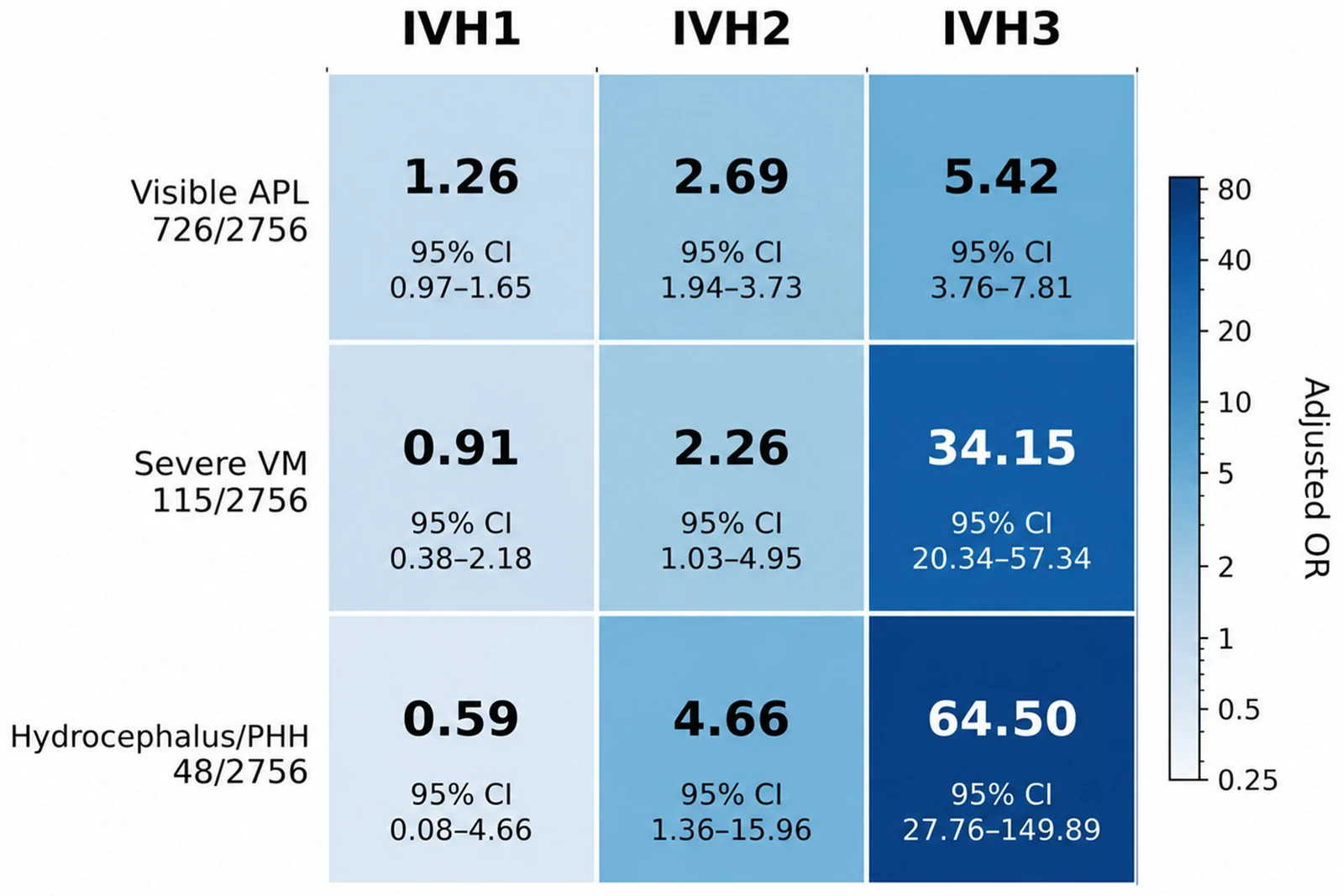
Adjusted associations of maximum IVH grade with visible APL, severe VM and hydrocephalus/PHH. Figure 2 shows adjusted odds ratios for visible APL, severe VM and hydrocephalus/PHH according to maximum IVH grade, using infants without IVH as the reference group. Models were adjusted for gestational age, sex and birth epoch. Severe VM was defined as maximum VM grade 3 across serial CUS. Hydrocephalus/PHH denotes clinically documented hydrocephalus during neonatal admission; in infants with IVH, hydrocephalus was considered post-haemorrhagic hydrocephalus. Darker shading indicates higher adjusted odds ratios. Abbreviations: APL, associated parenchymal lesion visible on serial CUS; CI, confidence interval; CUS, cranial ultrasound; IVH, intraventricular haemorrhage; PHH, post-haemorrhagic hydrocephalus; VM, ventriculomegaly.

After adjustment for GA, sex and birth epoch, the associations between maximum IVH grade and imaging phenotype components followed the same graded pattern (Figure 2; Supplementary Table S8). Compared with infants without IVH, IVH3 showed the largest adjusted associations, particularly for severe VM (aOR 34.15, 95% CI 20.34–57.34) and hydrocephalus/PHH (aOR 64.50, 95% CI 27.76–149.89), and was also associated with visible APL (aOR 5.42, 95% CI 3.76–7.81). Because visible APL ascertainment may have been influenced by changes in ultrasound technology and reporting practice, its distribution was examined by birth epoch (Supplementary Table S9). Visible APL among infants with IVH3 was broadly similar across epochs, whereas visible APL among infants with IVH2 was more frequent in 2011–2020. The composition of visible APL differed by maximum IVH grade (Supplementary Table S9). Among infants with IVH3 and visible APL, PVHI accounted for 46/92 (50.0%) cases, whereas transient bilateral periventricular echodensities resolving on follow-up were relatively more frequent in lower-grade IVH.

### 3.4. Developmental Outcomes in Isolated IVH and the Role of Severe VM

In the isolated-IVH analysis, infants with isolated IVH grade 1 or isolated IVH grade 2 had developmental outcome rates similar to those of infants without IVH and without visible APL, whereas isolated IVH grade 3 showed higher rates across all three developmental outcomes (Table 3). CP occurred in 1.4% of infants without IVH/no visible APL, 2.9% with isolated IVH grade 1, 2.8% with isolated IVH grade 2 and 27.5% with isolated IVH grade 3. School-age cognitive sequelae increased from 7.9% in infants without IVH/no visible APL to 9.5%, 13.6% and 24.3% across isolated IVH grades 1, 2 and 3, respectively. Sustained support increased from 18.5% to 19.3%, 32.4% and 47.5% across the same groups.

**Figure 3:**
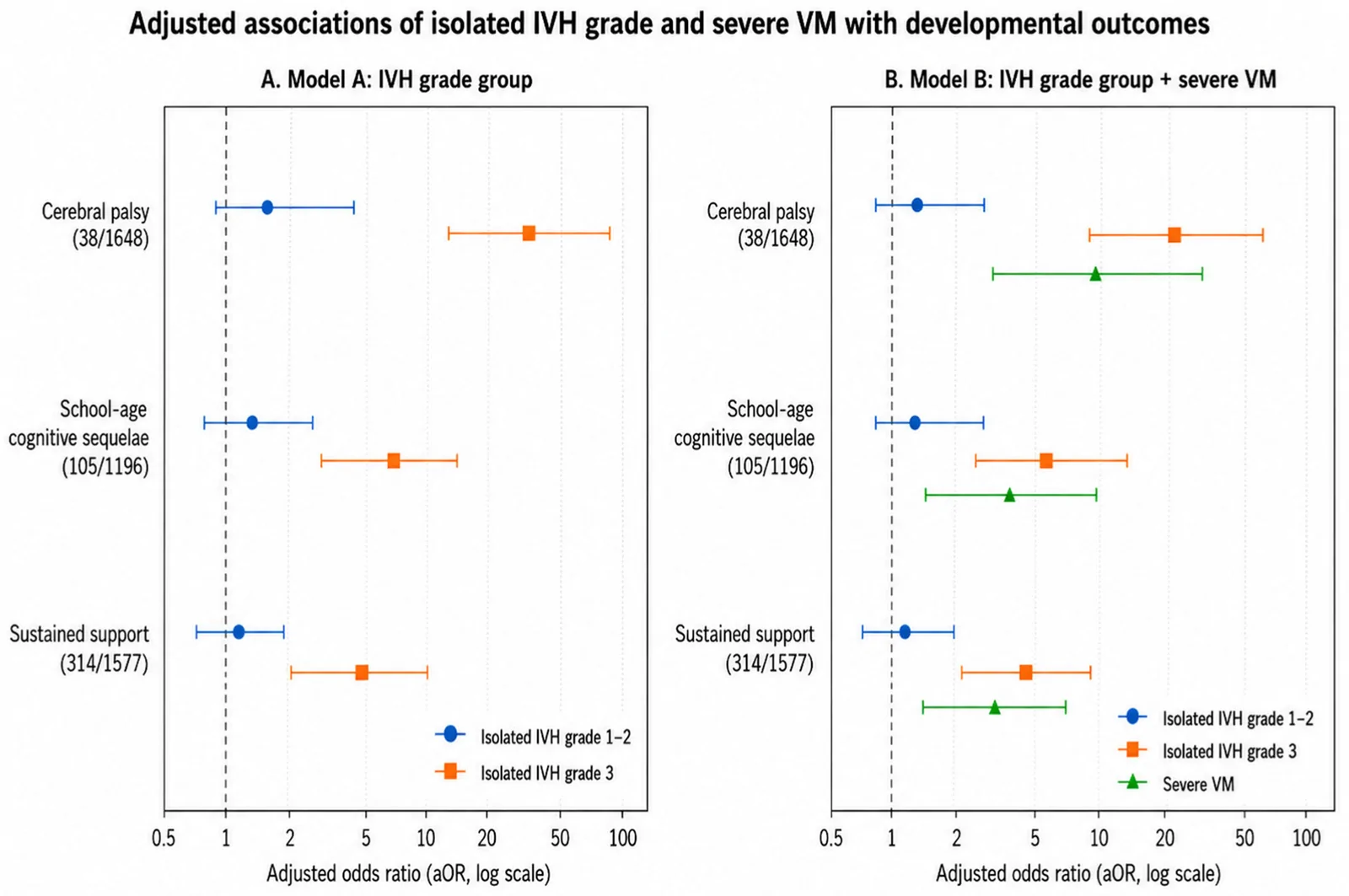
Adjusted associations of isolated IVH grade and severe VM with developmental outcomes. Figure 3 shows adjusted odds ratios for isolated IVH grade group and severe VM in the isolated-IVH analysis framework, using infants without IVH and without visible APL as the reference group. Model A included isolated IVH grade group and was adjusted for gestational age, sex and birth epoch. Model B additionally included severe VM. Severe VM was defined as maximum VM grade 3 across serial CUS. School-age cognitive sequelae refers to clinically classified school-age cognitive sequelae; sustained support refers to sustained developmental/educational support. Points represent aORs and horizontal lines indicate 95% confidence intervals. The vertical dashed line at aOR = 1.0 indicates the null value (no association). These models were not intended as formal mediation analyses. Abbreviations: APL, associated parenchymal lesion visible on serial CUS; aOR, adjusted odds ratio; CUS, cranial ultrasound; IVH, intraventricular haemorrhage; VM, ventriculomegaly.

In adjusted models, isolated IVH grade 1–2 showed no statistically significant association with CP, school-age cognitive sequelae or sustained support (Table 4; Figure 3). In contrast, isolated IVH grade 3 was associated with all three outcomes after adjustment for GA, sex and birth epoch, with the largest adjusted odds ratio observed for CP (aOR 28.55, 95% CI 11.31–72.11).

After inclusion of severe VM in the models, the adjusted odds ratio for isolated IVH grade 3 and CP decreased from 28.55 (95% CI 11.31–72.11) to 12.94 (95% CI 4.44–37.67) but remained statistically significant; severe VM was also associated with CP in the same model. For school-age cognitive sequelae and sustained support, the adjusted odds ratios for isolated IVH grade 3 decreased from 2.67 to 1.79 and from 2.88 to 1.77, respectively, and no longer reached statistical significance after inclusion of severe VM. Severe VM remained associated with both school-age cognitive sequelae and sustained support in these models (Table 4; Figure 3). In these mutually adjusted models, isolated IVH3 retained its clearest association with CP, whereas severe VM showed the clearer adjusted associations with school-age cognitive sequelae and sustained support.

Within isolated IVH grade 3, main developmental outcomes varied according to severe VM and PHH status, although these analyses were descriptive owing to small subgroup sizes (Table 5). Severe VM and PHH represented hierarchical rather than independent stratifications: all 9 infants with PHH were included among the 22 infants with severe VM, whereas 13 infants had severe VM without PHH. Compared with isolated IVH grade 3 without severe VM, isolated IVH grade 3 with severe VM showed higher observed frequencies of CP (33.3% vs. 22.7%) and sustained support (64.7% vs. 34.8%); school-age cognitive sequelae were similar in observed frequency (26.7% vs. 22.7%). The PHH subgroup was small (*n* = 9) and showed high observed frequencies of adverse outcomes, with CP in 3/7 (42.9%), school-age cognitive sequelae in 3/5 (60.0%) and sustained support in 4/6 (66.7%).

### 3.5. Secondary Analyses by CUS Phenotype and Parenchymal-Lesion Category

To contextualise the isolated-IVH analysis, secondary full CUS phenotype models compared infants across the six CUS phenotype groups (Supplementary Table S10). Compared with infants without IVH and without visible APL, infants with no IVH but visible APL had higher adjusted odds of CP (aOR 12.81, 95% CI 7.37–22.26), school-age cognitive sequelae (aOR 2.20, 95% CI 1.40–3.44) and sustained support (aOR 1.50, 95% CI 1.12–2.00). Infants with IVH and visible APL showed larger adjusted odds ratios, particularly for CP (aOR 25.64, 95% CI 14.39–45.71) and sustained support (aOR 3.04, 95% CI 2.16–4.27).

The distribution of CP type and severity across CUS phenotypes is shown in Supplementary Table S11. Among children with CP in the isolated IVH grade 3 group, spastic diplegia predominated (10/11, 90.9%); the remaining case was classified as other spastic-dystonic/ataxic CP (1/11, 9.1%). No hemiplegia or quadriplegia was recorded, and most cases were classified as GMFCS level I–II (9/11, 81.8%). In contrast, among children with CP in the IVH with visible APL group, hemiplegia was more frequent (22/52, 42.3%), spastic diplegia less frequent (12/52, 23.1%) and quadriplegia was recorded in 7/52 (13.5%).

Because visible APL was a broad composite used primarily to define whether IVH was isolated, secondary descriptive analyses examined CP frequency across refined parenchymal-lesion categories recorded on serial CUS (Supplementary Table S12). These analyses were intended to contextualise heterogeneity within visible APL rather than to provide validated lesion-specific prognostic estimates. Among infants with any IVH and visible APL, CP was recorded in 7/84 (8.3%) with transient bilateral periventricular echodensities resolving on follow-up, 3/25 (12.0%) with persistent or unresolved non-cystic periventricular echodensities and 42/85 (49.4%) with major visible parenchymal lesion. When the same refined categories were examined within the IVH3 subgroup, the corresponding CP frequencies were 1/11 (9.1%), 2/4 (50.0%) and 20/35 (57.1%), respectively. The IVH3-specific estimates were based on small denominators and should therefore be interpreted descriptively.

### 3.6. Sensitivity and Exploratory Analyses

Sensitivity and exploratory analyses were generally consistent with the primary models (Supplementary Tables S13–S17). Key findings were as follows:

- When moderate-to-severe VM (VM ≥ 2) was used instead of severe VM, associations were in the same direction but attenuated, particularly for CP (aOR 3.25, 95% CI 1.27–8.31, compared with 6.26, 95% CI 2.13–18.35 for severe VM; Table 4 and Supplementary Table S13).
- Exploratory hydrocephalus/PHH models showed large but imprecise associations, consistent with sparse events and wide confidence intervals. Hydrocephalus/PHH was associated with CP, school-age cognitive sequelae and sustained support, with adjusted odds ratios of 37.53 (95% CI 9.57–147.24), 20.57 (95% CI 4.78–88.55) and 4.63 (95% CI 1.36–15.78), respectively (Supplementary Table S14).
- Severe VM remained associated with sustained support among children without CP and without school-age cognitive sequelae (aOR 3.78, 95% CI 1.50–9.50; Supplementary Table S15). Firth penalised-likelihood logistic regression yielded results consistent with the primary CP models, with isolated IVH grade 3 and severe VM both remaining associated with CP (penalised aOR 12.45, 95% CI 4.39–35.32, and penalised aOR 6.16, 95% CI 2.17–17.53, respectively; Supplementary Table S16). Replacing GA with BW as the maturity covariate did not materially change the magnitude or direction of associations (Supplementary Table S17).

## 4. Discussion

In this large single-centre cohort of very preterm infants admitted to a tertiary NICU and undergoing protocolised serial CUS, the developmental significance of IVH was not captured by maximum haemorrhage grade alone. IVH behaved as a serial ultrasound phenotype composed of haemorrhage grade, visible APL on serial CUS, and ventricular trajectory. Higher maximum IVH grade was accompanied by increasing parenchymal and ventricular burden, with visible APL, severe VM and PHH present in 60.5%, 40.8% and 21.7% of infants with IVH3, respectively. When the analysis was restricted to isolated IVH, isolated grade 1–2 IVH showed low absolute CP rates, whereas isolated grade 3 IVH remained a clinically meaningful higher-risk phenotype (27.5% CP; 24.3% school-age cognitive sequelae; 47.5% sustained developmental/educational support). Severe VM further refined this risk: after its inclusion in adjusted models, associations between isolated IVH3 and several developmental outcomes were attenuated, while severe VM remained associated with CP, school-age cognitive sequelae and sustained developmental/educational support after adjustment. The CP phenotype after isolated IVH3 added neurological specificity, with predominance of spastic diplegia, absence of hemiplegia, and mostly mild functional severity. These findings support interpreting IVH in very preterm infants as a multidimensional serial CUS phenotype rather than by maximum grade alone.

This CUS-defined framework is inherently limited by ultrasound resolution. Isolated IVH in this study therefore denotes absence of a parenchymal lesion visible on serial CUS, not definitive absence of brain injury. We cannot exclude that some developmental findings after isolated IVH3, including the predominantly diplegic CP pattern, were partly attributable to punctate white-matter injury, small cerebellar haemorrhage or microstructural abnormalities not detectable by routine CUS [32].

The main imaging implication is that maximum IVH grade is not a pure descriptor of intraventricular blood burden. Higher grades carried greater likelihood of visible APL, severe VM and PHH, even after adjustment for GA, sex and birth epoch. This may help explain why outcome estimates based only on maximum IVH grade differ across studies. Contemporary reviews of germinal matrix–IVH and preterm brain injury increasingly emphasise that outcome depends on coexisting white-matter injury, PVHI, cerebellar haemorrhage, ventricular complications and secondary effects on brain maturation, rather than on haemorrhage grade alone [8,9,10,23,32]. The ELGAN 10-year CUS analysis is particularly relevant: by classifying infants according to combinations of IVH and white-matter damage, it showed that white-matter damage was the dominant ultrasound predictor of CP and cognitive impairment, whereas isolated IVH without white-matter damage was not clearly associated with these outcomes [20]. Our data extend this principle by documenting, across serial CUS windows, how frequently higher IVH grades, particularly IVH3, were accompanied by visible parenchymal and ventricular morbidity. Epoch-specific differences in visible APL frequency, particularly in IVH2, require caution: they may reflect changes in case-mix, survival, ultrasound resolution, acquisition practice or reporting thresholds, and should be interpreted as observed classification patterns rather than as unequivocal epidemiological trends.

This multidimensional interpretation is especially relevant to the controversy surrounding low-grade IVH. Meta-analyses and population-based cohorts have reported increased risks of CP, lower cognitive scores or school-age difficulties after grade 1–2 IVH [12,13,16,21,22], whereas other large cohorts have found modest or no clear association when major concomitant brain injury was absent [15,20]. A recent MRI-based study further showed that isolated low-grade IVH without concomitant white-matter injury or cerebellar haemorrhage was not significantly associated with adverse 3-year outcomes, whereas co-occurring parenchymal or cerebellar injury was [19]. Our findings suggest that these apparently conflicting results may partly reflect differences in the definition of “isolated” IVH, ascertainment of coexisting white-matter or cerebellar injury, use of MRI versus CUS, gestational-age case mix, outcome age and follow-up strategy. In the present cohort, IVH2 was heterogeneous: nearly half of infants with IVH2 had visible APL and a minority developed moderate-to-severe VM. In contrast, within the isolated IVH phenotype, CP occurred in only 2.9% of isolated IVH1 and 2.8% of isolated IVH2, close to the 1.4% observed in infants without IVH and without visible APL. The relatively reassuring interpretation therefore applies to isolated IVH1–2 as defined by serial CUS, not to low-grade IVH as a broad or incompletely phenotyped category.

Isolated IVH3 behaved differently from isolated IVH1–2. Even after excluding visible APL, isolated IVH3 retained a substantial developmental signal, with increased frequencies of CP, school-age cognitive sequelae and sustained developmental/educational support. A key contribution of this study is that it separates the IVH3 label from visible APL and then evaluates ventricular morbidity within the isolated IVH group. Severe VM and PHH identified subgroups with higher observed frequencies of adverse outcomes, although these analyses were descriptive because of small numbers. In adjusted models, adding severe VM attenuated the association between isolated IVH3 and CP from an aOR of 28.55 to 12.94, while severe VM was also associated with CP (aOR 6.26). For school-age cognitive sequelae and sustained support, associations with isolated IVH3 were attenuated after inclusion of severe VM, which retained the clearer adjusted associations with both outcomes. These mutually adjusted models should not be interpreted as formal mediation or causal inference; they suggest that developmental risk within isolated IVH3 is partly aligned with the post-haemorrhagic ventricular course. This interpretation is consistent with literature showing that ventricular size, progressive ventricular enlargement, hydrocephalus and need for intervention are clinically meaningful markers of later outcome [24,25,26,27,28,29,30,31]. The present analysis extends this literature by examining severe VM and PHH within isolated IVH3 rather than within mixed haemorrhagic–parenchymal injury.

The CP phenotype after isolated IVH3 provided additional neurological context. Spastic diplegia predominated, no hemiplegia was recorded, and most cases were functionally mild (GMFCS level I–II). This pattern contrasted with the IVH with visible APL group, in which hemiplegia was more frequent and quadriplegia occurred, and was more consistent with bilateral white-matter involvement than with focal unilateral PVHI [36,37,38]. Although occult injury detectable only by MRI cannot be excluded, systematic MRI would be required to distinguish occult parenchymal injury from altered white-matter maturation [39]. Intraventricular blood products may contribute to white-matter vulnerability through oxidative stress, inflammation, excitotoxicity and impaired oligodendrocyte maturation [23]. Severe VM may represent a marker of altered CSF dynamics, ventricular expansion, periventricular white-matter vulnerability or unmeasured co-occurring injury. Ventricular volume has been related to brain maturation and later neurodevelopment, and advanced MRI studies in infants with PHH have shown microstructural abnormalities involving periventricular white-matter tracts [28,40]. These mechanisms remain hypothetical, and the present observational analyses cannot determine whether ventricular dilatation contributed causally to developmental outcome.

The secondary full CUS phenotype and refined visible APL analyses reinforce why visible APL must be separated from isolated IVH. Infants with visible APL had substantially higher adjusted odds of CP, both without IVH and with IVH; the adjusted odds ratio for CP was 12.81 in infants with no IVH but visible APL and 25.64 in those with IVH and visible APL.

However, visible APL was not a homogeneous category. Transient bilateral periventricular echodensities resolving on follow-up had a much lower CP frequency than major visible parenchymal lesion, both among infants with any IVH and visible APL (7/84, 8.3% vs. 42/85, 49.4%) and within IVH3 (1/11, 9.1% vs. 20/35, 57.1%). Estimates for persistent or unresolved non-cystic periventricular echodensities were based on small denominators, particularly within IVH3. This is consistent with ultrasound-based white-matter injury classifications that distinguish transient echodensities from persistent, cystic or extensive lesions [32,35]. These subtype-specific comparisons contextualise the heterogeneity of the broad visible APL composite but should not be interpreted as a validated lesion-specific prognostic model. Systematic studies combining structured serial CUS classification with term-equivalent MRI are needed to clarify the prognostic significance of transient versus persistent non-cystic periventricular echodensities in infants with IVH [39].

The school-age and support outcomes broaden the interpretation beyond CP. CP is a robust and clinically meaningful motor outcome, but it does not capture all later developmental vulnerability in very preterm children. High-grade IVH has been associated with later cognitive, academic and executive difficulties, and recent population-based data show persistently lower school performance after high-grade IVH compared with very preterm controls without IVH [10,41]. In the present cohort, sustained developmental/educational support increased after isolated grade 3 IVH and remained more frequent in exploratory analyses among children without CP and without clinically classified school-age cognitive sequelae. This suggests that sustained support captured a broader functional vulnerability pathway not fully represented by binary CP or cognitive sequelae variables. This interpretation is consistent with recent umbrella-review evidence showing that preterm birth and low BW are associated with persistent cognitive and educational disadvantages across the life course, with greater vulnerability at lower GAs and lower BWs [42]. Nevertheless, sustained developmental/educational support should not be interpreted as a formal neuropsychological endpoint, because it is influenced by referral pathways, school resources, local educational policy and changes in practice across epochs. Its value in this study is as a pragmatic paediatric indicator of sustained functional need.

This study has several strengths. It used a large single-centre tertiary NICU cohort with protocolised serial CUS across three decades, allowing IVH to be described longitudinally rather than as a single maximum grade. Maximum IVH grade and maximum VM grade were defined across available serial CUS windows using the same rules for all infants. The main contribution of the study is the integration of three serial CUS dimensions that are often considered separately: maximum IVH grade, presence or absence of visible APL, and ventricular morbidity. By separating IVH from visible APL, stratifying isolated IVH by maximum grade, and assessing severe VM and hydrocephalus/PHH as ventricular risk-refining variables, the present analysis supports a more granular bedside interpretation of IVH in very preterm infants. The CP type and severity analysis also added neurological specificity to the interpretation of isolated IVH3.

Several limitations should be acknowledged. First, the developmental analyses were affected by survivor and ascertainment conditioning. Inclusion in the main serial CUS cohort required survival and clinical stability long enough to undergo at least two complete CUS examinations, and follow-up analyses additionally required survival and available developmental information. This limits generalisability to the sickest and most immature infants and may underestimate the total burden associated with severe IVH phenotypes when early death precluded serial imaging or developmental assessment. Later-window CUS availability was also lower in infants with higher maximum IVH grade and in higher-severity CUS phenotypes, indicating differential imaging opportunity. Missing CUS windows were treated as unavailable and were not imputed as normal; nevertheless, incomplete late imaging may have influenced ascertainment of evolving ventricular dilatation or later parenchymal findings.

Second, the study spans three decades, during which neonatal care, survival at lower GAs, ultrasound technology, acquisition practice, reporting terminology and follow-up pathways changed. Birth epoch was included in adjusted models, but residual secular effects may remain. Imaging classification was based on contemporaneous routine clinical ultrasound reports recorded in the neonatal registry rather than systematic blinded re-review of stored images, and interobserver reliability was not assessed. Systematic MRI was not available across the full cohort or used to define the primary phenotypes; therefore, isolated IVH denotes absence of visible APL on serial CUS rather than definitive absence of subtle parenchymal, cerebellar or microstructural injury. Other limitations relate to outcomes and modelling. Clinically classified school-age cognitive sequelae were assigned from heterogeneous longitudinal clinical, neuropsychological and educational sources and should be interpreted as pragmatic clinical follow-up outcomes rather than harmonised psychometric endpoints. Sustained developmental/educational support was also a pragmatic functional indicator and may have been influenced by referral pathways, local educational resources and changes in practice across epochs. Follow-up availability differed across outcomes and phenotype groups, and available-case analyses may therefore be affected by non-random missingness. Subgroup analyses within isolated IVH3, hydrocephalus/PHH and CP phenotype were based on small numbers and should be interpreted descriptively. Penalised logistic regression sensitivity analyses supported the main CP models, and replacing GA with BW produced similar conclusions, but all models remain descriptive and observational. The adjusted models used a parsimonious covariate set focused on GA, sex and birth epoch; they were not intended to estimate the causal effect of IVH independent of the broader neonatal course. Postnatal morbidities and treatments were not included in the primary models because they may occur after the imaging exposure, may lie on the pathway between prematurity, brain injury and developmental outcome, and may not have been uniformly recorded or comparable across epochs. The findings therefore support associations relevant to developmental risk stratification, not causal inference or formal prediction.

Clinically, these findings suggest potential value in reporting IVH as a multidimensional serial CUS phenotype incorporating maximum IVH grade, visible APL and ventricular course. Isolated IVH1–2 showed low absolute CP risk within the limits of CUS ascertainment, whereas isolated IVH3 may warrant closer attention to ventricular evolution and developmental surveillance. Future multicentre studies should combine protocolised serial CUS with systematic term-equivalent MRI, quantitative ventricular measures and harmonised school-age neuropsychological assessment to validate this framework and clarify the mechanisms underlying developmental outcomes after isolated IVH3.

## 5. Conclusions

Maximum IVH grade remains an important first layer of neonatal neurodevelopmental risk stratification, but it should not be interpreted in isolation. In this single-centre retrospective cohort, serial CUS allowed IVH to be characterised by haemorrhage grade, visible APL status and ventricular course, thereby distinguishing isolated grade 1–2 IVH from isolated grade 3 IVH and from IVH accompanied by visible parenchymal injury. Isolated grade 1–2 IVH showed low absolute CP risk, whereas isolated grade 3 IVH remained a clinically meaningful higher-risk phenotype, particularly when accompanied by severe VM or hydrocephalus/PHH. These findings suggest that structured serial CUS reporting may support bedside counselling and targeted follow-up planning, but external validation in cohorts with systematic MRI and harmonised neurodevelopmental assessment is needed before broad implementation.

## Figures and Tables

**Table 1:** Perinatal and neonatal characteristics by cranial ultrasound phenotype.

Characteristic	No IVH, No Visible APL	No IVH + Visible APL	Isolated IVH1	Isolated IVH2	Isolated IVH3	IVH + Visible APL	*p* Value
*N*	1617	446	259	94	60	280	—
Gestational age, median (IQR), weeks	30 (28, 31)	29 (27, 31)	29 (27, 31)	26 (25, 28)	27 (25, 28)	27 (25, 29)	<0.001
Birth weight, median (IQR), g	1200 (953, 1395)	1150 (862, 1350)	1150 (900, 1385)	870 (702, 1138)	1008 (795, 1196)	990 (780, 1232)	<0.001
Male sex	809/1617 (50.0%)	221/446 (49.6%)	149/259 (57.5%)	52/94 (55.3%)	36/60 (60.0%)	168/280 (60.0%)	0.007
Multiple gestation	540/1617 (33.4%)	147/446 (33.0%)	68/259 (26.3%)	22/94 (23.4%)	17/60 (28.3%)	60/280 (21.4%)	<0.001
5 min Apgar score, median (IQR)	9 (8, 10)	8 (7, 9)	9 (7, 10)	7 (6, 9)	7 (5, 8)	7 (6, 9)	<0.001
Birth epoch: 1991–2000	441/1617 (27.3%)	110/446 (24.7%)	76/259 (29.3%)	12/94 (12.8%)	17/60 (28.3%)	61/280 (21.8%)	<0.001
Birth epoch: 2001–2010	545/1617 (33.7%)	70/446 (15.7%)	81/259 (31.3%)	35/94 (37.2%)	27/60 (45.0%)	74/280 (26.4%)	—
Birth epoch: 2011–2020	631/1617 (39.0%)	266/446 (59.6%)	102/259 (39.4%)	47/94 (50.0%)	16/60 (26.7%)	145/280 (51.8%)	—
Death before discharge	61/1617 (3.8%)	29/446 (6.5%)	23/259 (8.9%)	12/94 (12.8%)	11/60 (18.3%)	60/280 (21.4%)	<0.001
Survival to discharge	1556/1617 (96.2%)	417/446 (93.5%)	236/259 (91.1%)	82/94 (87.2%)	49/60 (81.7%)	220/280 (78.6%)	<0.001

Values are *n*/*N* (%), median (IQR), or as otherwise stated. APL denotes associated parenchymal lesion visible on serial CUS. *p* values are descriptive and compare CUS phenotype groups. Isolated IVH was defined as IVH grade 1–3 without visible APL; VM and hydrocephalus/PHH were not used to define isolation. Death before discharge denotes all deaths occurring before neonatal discharge. Abbreviations: APL, associated parenchymal lesion; CUS, cranial ultrasound; IQR, interquartile range; IVH, intraventricular haemorrhage; PHH, post-haemorrhagic hydrocephalus; VM, ventriculomegaly.

**Table 2:** Parenchymal lesions, ventriculomegaly and hydrocephalus across maximum IVH grade categories.

Maximum IVH Grade	*N*	Visible APL	No Visible APL	VM 0–1	VM 2	VM 3	Hydrocephalus/PHH
No IVH	2063	446/2063 (21.6%)	1617/2063 (78.4%)	1942/2063 (94.1%)	83/2063 (4.0%)	38/2063 (1.8%)	10/2063 (0.5%)
Grade 1 IVH	354	95/354 (26.8%)	259/354 (73.2%)	332/354 (93.8%)	16/354 (4.5%)	6/354 (1.7%)	1/354 (0.3%)
Grade 2 IVH	187	93/187 (49.7%)	94/187 (50.3%)	152/187 (81.3%)	26/187 (13.9%)	9/187 (4.8%)	4/187 (2.1%)
Grade 3 IVH	152	92/152 (60.5%)	60/152 (39.5%)	55/152 (36.2%)	35/152 (23.0%)	62/152 (40.8%)	33/152 (21.7%)
*p* value	—	<0.001	<0.001	<0.001	<0.001	<0.001	<0.001

Values are *n*/*N* (%), unless otherwise stated. Maximum IVH grade was defined as the highest IVH grade recorded across serial CUS. APL denotes associated parenchymal lesion visible on serial CUS and was defined according to the registry-based study definition. Severe VM was defined as maximum VM grade 3 across serial CUS. Hydrocephalus/PHH denotes clinically documented hydrocephalus during neonatal admission; in infants with IVH, hydrocephalus was considered post-haemorrhagic hydrocephalus (PHH). This table describes how increasing IVH grade was accompanied by visible parenchymal and ventricular burden; it was not intended to estimate causal pathways. *p* values are descriptive tests of association across maximum IVH grade groups. Abbreviations: APL, associated parenchymal lesion; CUS, cranial ultrasound; IVH, intraventricular haemorrhage; PHH, post-haemorrhagic hydrocephalus; VM, ventriculomegaly.

**Table 3:** Cerebral palsy, cognitive and functional outcomes in isolated IVH.

Group	*N*	Cerebral Palsy	Clinically Classified School-Age Cognitive Sequelae	Sustained Developmental/Educational Support
No IVH, no visible APL	1617	19/1331 (1.4%)	76/968 (7.9%)	236/1277 (18.5%)
Isolated IVH1	259	6/206 (2.9%)	14/147 (9.5%)	37/192 (19.3%)
Isolated IVH2	94	2/71 (2.8%)	6/44 (13.6%)	22/68 (32.4%)
Isolated IVH3	60	11/40 (27.5%)	9/37 (24.3%)	19/40 (47.5%)
*p* value	—	<0.001	0.003	<0.001

Values are *n*/*N* (%). Isolated IVH was defined as IVH grade 1–3 without associated parenchymal lesion visible on serial CUS. The descriptive comparator group was infants without IVH and without visible APL. Denominators vary because outcome availability differed across follow-up domains. Clinically classified school-age cognitive sequelae refers to cognitive sequelae recorded from longitudinal clinical, neurodevelopmental and educational follow-up sources around school age. Sustained developmental/educational support included support starting before 2 years and maintained thereafter, preschool support maintained thereafter, or support first recorded at primary-school age. *p* values are descriptive overall comparisons across groups. Abbreviations: APL, associated parenchymal lesion;CUS, cranial ultrasound; IVH, intraventricular haemorrhage.

**Table 4:** Adjusted associations of isolated IVH grade and severe VM with main developmental outcomes.

Outcome	Model	Events/*N*	Isolated IVH1–2 aOR (95% CI)	*p*	Isolated IVH3 aOR (95% CI)	*p*	Severe VM aOR (95% CI)	*p*
Cerebral palsy	Model A: IVH grade group	38/1648	2.12 (0.90–4.99)	0.085	28.55 (11.31–72.11)	<0.001	—	—
Cerebral palsy	Model B: IVH grade group + severe VM	38/1648	2.07 (0.87–4.90)	0.098	12.94 (4.44–37.67)	<0.001	6.26 (2.13–18.35)	<0.001
Clinically classified school-age cognitive sequelae	Model A: IVH grade group	105/1196	1.18 (0.69–2.01)	0.542	2.67 (1.17–6.12)	0.020	—	—
Clinically classified school-age cognitive sequelae	Model B: IVH grade group + severe VM	105/1196	1.15 (0.67–1.97)	0.608	1.79 (0.70–4.58)	0.227	2.54 (1.05–6.15)	0.038
Sustained developmental/educational support	Model A: IVH grade group	314/1577	1.12 (0.80–1.56)	0.518	2.88 (1.48–5.58)	0.002	—	—
Sustained developmental/educational support	Model B: IVH grade group + severe VM	314/1577	1.10 (0.79–1.54)	0.582	1.77 (0.85–3.70)	0.130	3.40 (1.70–6.77)	<0.001

Reference group: infants without IVH and without associated parenchymal lesion visible on serial CUS. Model A included isolated IVH grade group and was adjusted for gestational age, sex and birth epoch. Model B included isolated IVH grade group and severe VM and was adjusted for gestational age, sex and birth epoch. Severe VM was defined as maximum VM grade 3 across serial CUS. These models were not intended as formal mediation analyses. Abbreviations: aOR, adjusted odds ratio; CI, confidence interval; CUS, cranial ultrasound; IVH, intraventricular haemorrhage; VM, ventriculomegaly.

**Table 5:** Developmental outcomes within isolated IVH grade 3 stratified by severe VM and post-haemorrhagic hydrocephalus.

Subgroup	Subgroup *N*	Cerebral Palsy	Clinically Classified School-Age Cognitive Sequelae	Sustained Developmental/Educational Support
**Stratification by severe VM**				
Isolated IVH3 without severe VM	38	5/22 (22.7%)	5/22 (22.7%)	8/23 (34.8%)
Isolated IVH3 with severe VM	22	6/18 (33.3%)	4/15 (26.7%)	11/17 (64.7%)
**Stratification by hydrocephalus/PHH**				
Isolated IVH3 without PHH	51	8/33 (24.2%)	6/32 (18.8%)	15/34 (44.1%)
Isolated IVH3 with PHH	9	3/7 (42.9%)	3/5 (60.0%)	4/6 (66.7%)

Values are *n*/*N* (%). This descriptive analysis was restricted to infants with isolated IVH grade 3, defined as IVH grade 3 without visible APL on serial CUS. Severe VM and hydrocephalus/PHH were hierarchically related, and the two stratifications were not independent. All 9 infants with hydrocephalus/PHH were included among the 22 infants with severe VM; 13 infants had severe VM without hydrocephalus/PHH. Severe VM was defined as maximum VM grade 3 across serial CUS. Hydrocephalus was defined as progressive ventricular dilatation on serial CUS accompanied by clinical signs suggestive of raised intracranial pressure and/or the need for temporising or definitive cerebrospinal-fluid diversion; in infants with IVH, hydrocephalus was considered post-haemorrhagic hydrocephalus (PHH). Non-dilative intracranial hypertension was not classified as PHH. Ventricular status was not used to define isolated IVH. Analyses were descriptive because of small subgroup sizes. School-age cognitive sequelae refers to clinically classified school-age cognitive sequelae; sustained support refers to sustained developmental/educational support. Abbreviations: APL, associated parenchymal lesion visible on serial CUS; CUS, cranial ultrasound; IVH, intraventricular haemorrhage; PHH, post-haemorrhagic hydrocephalus; VM, ventriculomegaly.

## Data Availability

The data presented in this study are available on reasonable request from the corresponding author. The data are not publicly available because they derive from a clinical registry of very preterm infants and contain sensitive health information subject to institutional, ethical and privacy restrictions. Any data sharing would require appropriate institutional approval and compliance with applicable data protection regulations.

## Supplementary Materials

The following supporting information can be downloaded at https://www.mdpi.com/article/10.3390/jcm15166310/s1: Supplementary Methods S1–S8 and Supplementary Tables S1–S18. Supplementary Methods include cohort assembly, eligibility criteria, birth epochs, CUS window definitions, CUS reporting source, imaging phenotype definitions, ventriculomegaly and hydrocephalus/PHH definitions, neurodevelopmental follow-up procedures and supplementary statistical analyses. Supplementary Tables include CUS availability, exclusion reasons, follow-up availability, missingness patterns, imaging phenotype analyses, sensitivity analyses and additional developmental outcome analyses. Method S1: Study cohort, eligibility criteria, birth epochs and cohort assembly; Method S2: Serial CUS protocol, window selection, completeness criteria and missing imaging data; Method S3: CUS reporting source, image interpretation, ultrasound technology and MRI availability; Method S4: IVH grading, visible APL definition and refined parenchymal-lesion categories; Method S5: Ventriculomegaly grading and hydrocephalus/PHH definitions; Method S6: Full CUS phenotype framework; Method S7: Neurodevelopmental follow-up programme, outcome definitions and missingness; Method S8: Supplementary statistical analyses; Table S1: Cranial ultrasound availability, cohort assembly and imaging completeness in the study cohort; Table S2: Baseline characteristics of infants included versus excluded from the main serial CUS analysis; Table S3: Developmental follow-up availability by CUS phenotype; Table S4: Missingness patterns for developmental follow-up outcomes; Table S5: Maximum IVH grade by gestational age and birth epoch; Table S6: Temporal pattern of grade 2 and grade 3 IVH; Table S7: Timing of maximum IVH grade; Table S8: Adjusted associations between maximum IVH grade and imaging phenotype components; Table S9: Associated parenchymal lesions visible on serial CUS according to IVH and birth epoch; Table S10: Adjusted associations of full CUS phenotype with clinical outcomes; Table S11: Cerebral palsy type and severity according to CUS phenotype; Table S12: Cerebral palsy according to refined visible APL category; Table S13: Sensitivity models using moderate-to-severe VM (VM ≥ 2) instead of severe VM; Table S14: Exploratory models with hydrocephalus/PHH; Table S15: Sustained developmental/educational support analyses in the isolated-IVH framework; Table S16: Firth penalised-likelihood logistic regression sensitivity analyses for cerebral palsy; Table S17: Birth-weight-adjusted sensitivity analysis replacing gestational age in primary adjusted models; Table S18: Ventriculomegaly severity mapping used in the study. References [43,44,45] are cited in Supplementary Materials.

## Abbreviations

The following abbreviations are used in this manuscript:

aOR	adjusted odds ratio
APL	associated parenchymal lesion
BW	birth weight
CEIm	Research Ethics Committee for Medicines
CI	confidence interval
CP	cerebral palsy
CUS	cranial ultrasound
GA	gestational age
GMFCS	Gross Motor Function Classification System
IQ	intelligence quotient
IVH	intraventricular haemorrhage
IVH1	grade 1 intraventricular haemorrhage
IVH2	grade 2 intraventricular haemorrhage
IVH3	grade 3 intraventricular haemorrhage
MRI	magnetic resonance imaging
NICU	neonatal intensive-care unit
PHH	post-haemorrhagic hydrocephalus
PVHI	periventricular haemorrhagic infarction
TEA	term-equivalent age
VM	ventriculomegaly
