# Supplementary material for: Beyond Maximum Haemorrhage Grade: Serial Cranial Ultrasound Phenotyping Refines Developmental Risk Stratification After Intraventricular Haemorrhage in Very Preterm Infants
Source: J Clin Med. 2026 Aug 14;15(16):6310. doi: 10.3390/jcm15166310 (PMC13513188; doi:10.3390/jcm15166310)
Supplement: Supplementary file 1 [file jcm-15-06310-s001.zip › jcm-4416773-supplementary.pdf]

Supplementary Materials

Beyond Maximum Haemorrhage Grade: Serial Cranial Ultrasound Phenotyping Refines Developmental Risk Stratification After Intraventricular Haemorrhage in Very Preterm Infants

Supplementary Methods

- Method S1. Study cohort, eligibility criteria, birth epochs and cohort assembly
- Method S2. Serial CUS protocol, window selection, completeness criteria and missing imaging data
- Method S3. CUS reporting source, image interpretation, ultrasound technology and MRI availability
- Method S4. IVH grading, visible APL definition and refined parenchymal-lesion categories
- Method S5. Ventriculomegaly grading and hydrocephalus/PHH definitions
- Method S6. Full CUS phenotype framework
- Method S7. Neurodevelopmental follow-up programme, outcome definitions and missingness
- Method S8. Supplementary statistical analyses

Supplementary Tables index

Table	Title
S1	Cranial ultrasound availability, cohort assembly and imaging completeness in the study cohort
Panel A	CUS availability by birth epoch
Panel B	Reasons for exclusion from the main serial CUS cohort
Panel C	CUS availability by maximum IVH grade within the main serial CUS cohort
Panel D	CUS availability by CUS phenotype within the main serial CUS cohort
S2	Baseline characteristics of infants included versus excluded from the main serial CUS analysis
S3	Developmental follow-up availability by CUS phenotype
S4	Missingness patterns for developmental follow-up outcomes
Panel A	Missingness pattern for cerebral palsy follow-up
Panel B	Missingness pattern for clinically classified school-age cognitive sequelae
Panel C	Missingness pattern for sustained developmental/educational support
S5	Maximum IVH grade by gestational age and birth epoch
S6	Temporal pattern of grade 2 and grade 3 IVH
S7	Timing of maximum IVH grade
S8	Adjusted associations between maximum IVH grade and imaging phenotype components
S9	Associated parenchymal lesions visible on serial CUS according to IVH and birth epoch
Panel A	Associated parenchymal lesion visible on serial CUS by birth epoch and maximum IVH grade
Panel B	Composition of associated parenchymal lesion visible on serial CUS by IVH status and maximum IVH grade
S10	Adjusted associations of full CUS phenotype with clinical outcomes
S11	Cerebral palsy type and severity according to CUS phenotype
S12	Cerebral palsy according to refined visible APL category
S13	Sensitivity models using moderate-to-severe VM (VM≥2) instead of severe VM
S14	Exploratory models with hydrocephalus/PHH
S15	Sustained developmental/educational support analyses in the isolated-IVH framework
Panel A	Sustained developmental/educational support among children without cerebral palsy and/or school-age cognitive sequelae
Panel B	Exploratory adjusted association between severe VM and sustained developmental/educational support
S16	Firth penalised-likelihood logistic regression sensitivity analyses for cerebral palsy

Table	Title
S1	Cranial ultrasound availability, cohort assembly and imaging completeness in the study cohort
S17	Birth-weight-adjusted sensitivity analysis replacing gestational age in primary adjusted models
S18	Ventriculomegaly severity mapping used in the study

Method S1. Study cohort, eligibility criteria, birth epochs and cohort assembly

Eligible infants were live-born very preterm infants, defined as <32 weeks' gestational age and/or birth weight ≤1,500 g, delivered at the study centre or admitted within the first 48 h of life between 1991 and 2020. The source cohort comprised 3,081 infants. The main serial CUS cohort included infants with at least two complete CUS examinations, where a complete CUS window required availability of IVH grade, VM grade and parenchymal lesion pattern.

Birth epoch was categorised as 1991–2000, 2001–2010 and 2011–2020 to account for secular changes in neonatal care, survival, ultrasound technology, reporting practice and follow-up pathways. The cohort spans three decades, during which neonatal care and survival at lower gestational ages changed substantially. Changes included progressive implementation of antenatal corticosteroids, surfactant therapy, non-invasive respiratory support, refined ventilation strategies, infection-control practices, nutritional support and family-centred developmental care.

The four-window CUS surveillance framework and the three-domain registry structure were maintained across the study period, although ultrasound equipment, image quality and reporting detail evolved. Birth epoch was therefore included as a prespecified covariate in adjusted models. Infants without sufficient serial CUS data were described separately to quantify the degree to which developmental outcome analyses were conditioned on survival and imaging availability.

Method S2. Serial CUS protocol, window selection, completeness criteria and missing imaging data

Routine cranial ultrasound surveillance was summarised using four prespecified windows: CUS1, within the first 48 h after birth; CUS7, around day 7; CUS28, around day 28; and discharge/term-equivalent age (TEA), near term-equivalent age or discharge, whichever occurred first. For the present analysis, each window was represented by a single registry-recorded surveillance assessment. If more than one CUS examination occurred within a given window, the protocol-defined assessment used for registry surveillance was retained. If no complete assessment was available for a given window, that window was coded as unavailable rather than normal. A CUS window was considered complete when IVH grade, VM grade and parenchymal lesion pattern were all available. Incomplete windows were not used to define complete-window longitudinal phenotypes. Missing imaging windows were not imputed. This approach was chosen to avoid misclassifying unavailable scans as normal and to preserve the distinction between absence of recorded abnormality and absence of imaging opportunity.

Method S3. CUS reporting source, image interpretation, ultrasound technology and MRI availability

CUS examinations were performed as part of routine neonatal intensive-care imaging and reported in clinical practice by the paediatric radiology team, including senior paediatric radiologists. For this study, CUS findings were extracted from contemporaneous routine clinical reports recorded in the neonatal registry rather than from post hoc blinded re-review of stored images.

Systematic blinded re-review of stored images was not performed, and interobserver reliability was not assessed. Ultrasound machines, probes, image resolution and reporting detail changed over the three-decade study period. These temporal changes may have influenced detection and reporting of subtle or transient lesions, particularly non-cystic periventricular echodensities.

MRI was available in a subset of infants, but availability was not uniform across birth epochs, survival status or clinical groups and MRI was not used to define the primary imaging phenotypes. In earlier epochs, MRI was obtained more selectively. Consequently, isolated IVH in this study refers to IVH without visible APL on serial CUS, not to the definitive absence of subtle parenchymal, cerebellar or microstructural injury detectable only by MRI.

Method S4. IVH grading, visible APL definition and refined parenchymal-lesion categories

At each selected CUS window, three domains were recorded: intraventricular haemorrhage (IVH), ventriculomegaly (VM) and parenchymal lesion pattern.

- **IVH** was classified as absent or graded from 1 to 3 according to the Papile classification as modified by Volpe [33,34], in which periventricular haemorrhagic infarction (PVHI) is recognised as a parenchymal venous infarction and is therefore analysed separately as an associated parenchymal lesion rather than as an additional IVH grade. In bilateral cases, the highest grade recorded on either side was used. Maximum IVH grade was defined as the highest grade recorded across available serial CUS windows.
- **The parenchymal-lesion grading** component used an institutional 0–4 ultrasound scale adapted from de Vries and colleagues [35]. LP1 and LP2 corresponded to non-cystic echogenic abnormalities <1 cm and ≥1 cm, LP3 to cystic white-matter injury <1 cm, and LP4 to cystic white-matter injury ≥1 cm or extensive cystic/multifocal parenchymal injury, according to the registry scale. We used the term "associated parenchymal lesion visible on serial CUS" (visible APL) as a study-specific registry-derived composite variable to distinguish isolated IVH from IVH accompanied by any parenchymal abnormality detected on routine serial CUS.
- **Visible APL** included PVHI, cystic white-matter injury, non-cystic periventricular echodensities, arterial infarction, parenchymal haemorrhage, deep grey matter lesion, cerebellar lesion, multicystic encephalomalacia and mixed or other major focal/structural abnormalities recorded

in the neonatal registry. This broad composite was used primarily to determine whether IVH occurred without any parenchymal abnormality visible on serial CUS and thereby to define isolated IVH; it was not intended to imply biological or prognostic homogeneity across lesion subtypes. Because visible APL grouped lesions with different pathophysiology, laterality and prognostic weight, individual visible APL subtypes were retained for descriptive and secondary analyses.

For secondary analyses, visible APL was further grouped into refined categories: transient bilateral periventricular echodensities resolving on follow-up; persistent or unresolved non-cystic periventricular echodensities; and major visible parenchymal lesion, including PVHI, cystic white-matter injury, arterial infarction, parenchymal haemorrhage, deep grey matter or cerebellar lesion, multicystic encephalomalacia, mixed lesions or other major focal/structural abnormalities. These refined analyses were intended to contextualise heterogeneity within the broad visible APL composite rather than to provide validated lesion-specific prognostic estimates.

Method S5. Ventriculomegaly grading and hydrocephalus/PHH definitions

Severe VM and hydrocephalus/post-haemorrhagic hydrocephalus (PHH) were treated as hierarchically related but analytically distinct variables. VM was defined as an imaging finding reflecting ventricular size and morphology on CUS and was graded at each selected CUS window according to the registry-based institutional 0–3 scale. Maximum VM grade was defined as the highest VM grade recorded across available serial CUS windows. Severe VM was defined as maximum VM grade 3 and was selected as the main ventricular trajectory variable. Moderate-to-severe VM was defined as maximum VM grade ≥2 and was examined in sensitivity analyses.

The institutional VM grading scale was based on long-standing neonatal CUS practice and was benchmarked against historical and contemporary reference standards for neonatal ventricular measurements, including the Levene ventricular index, atrial width, thalamo-occipital distance and published reference ranges [43–45]. The ventriculomegaly severity mapping used in the study is provided in Supplementary Table S18.

- **Thalamo-occipital distance** was defined as the parasagittal inner-to-inner linear distance from the posterior thalamic margin to the inner border of the occipital horn of the lateral ventricle. Atrial width was defined as the axial transventricular inner-to-inner diameter at the ventricular atrium. Acquisition was performed on a true axial transventricular plane through the atrium; oblique planes were avoided when measurements were available. Because the cohort spans three decades, raw quantitative ventricular measurements were not uniformly available for every scan and stored images were not

systematically remeasured. The registry VM grade therefore represents the recorded clinical ultrasound severity category, supported by available measurements when documented.

- **Hydrocephalus** was defined according to institutional practice as progressive ventricular dilatation on serial CUS accompanied by clinical signs of raised intracranial pressure and/or the need for temporising or definitive cerebrospinal-fluid diversion. In infants with IVH, hydrocephalus was considered post-haemorrhagic hydrocephalus (PHH). All infants with PHH met criteria for severe VM, whereas severe VM could occur without clinically documented PHH. PHH therefore represented a clinicoradiologically defined subset of severe VM rather than an independent or mutually exclusive ventricular phenotype. Non-dilative intracranial hypertension was not classified as PHH. Hydrocephalus/PHH was analysed as an exploratory clinical-course variable reflecting the most severe post-haemorrhagic ventricular complication. The frontal-occipital horn ratio was not routinely available across the three-decade cohort and was therefore not used to define VM grade or hydrocephalus/PHH.

Method S6. Full CUS phenotype framework

The full CUS phenotype was defined using maximum IVH grade and the presence or absence of visible APL on serial CUS. Six mutually exclusive phenotype groups were used: no IVH/no visible APL; no IVH with visible APL; isolated grade 1 IVH (IVH1); isolated grade 2 IVH (IVH2); isolated grade 3 IVH (IVH3); and IVH with visible APL.

Isolated IVH was defined as IVH grade 1–3 without visible APL. This definition did not exclude VM or hydrocephalus/PHH, because post-haemorrhagic ventricular trajectory was one of the main components under study. VM and hydrocephalus/PHH were analysed separately as ventricular morbidity variables.

Maximum IVH grade was analysed as the highest IVH grade recorded across available serial CUS windows. Severe ventricular trajectory was primarily defined as severe VM, corresponding to maximum VM grade 3 across serial CUS. Moderate-to-severe VM (VM≥2) and hydrocephalus/PHH were examined in sensitivity or exploratory analyses.

Method S7. Neurodevelopmental follow-up programme, outcome definitions and missingness

Neurodevelopmental follow-up was part of the institutional longitudinal programme for very preterm infants. Children were assessed from infancy through early childhood and, when available or clinically indicated, around the 7-year follow-up window. Follow-up included neurological examination, assessment of motor and developmental progress, review of cognitive and educational functioning, and documentation of ongoing therapeutic, developmental or educational support. Children with cerebral palsy (CP), clinically classified school-age cognitive sequelae or other relevant neurological morbidity could remain under follow-up beyond the standard programme according to clinical need.

- **CP** was primarily assessed around 2 years' corrected age and was analysed as a binary survivor outcome. Among children with CP, clinical type and functional severity were summarised descriptively. Functional severity was grouped using Gross Motor Function Classification System (GMFCS)-equivalent levels as ambulatory CP for levels I–II and non-ambulatory CP for levels III–V. CP type was derived from the registry CP motor-type variable when available and grouped descriptively as hemiplegia, spastic diplegia, quadriplegia or other/mixed CP, including spastic-dystonic and ataxic forms. These categories reflected the available registry classification; no retrospective reclassification according to the Surveillance of Cerebral Palsy in Europe framework was attempted. Type-specific analyses were descriptive only because subgroup sizes were small. Denominators for CP analyses excluded survivors without available CP status.
- **Assessment of cognition and educational functioning** was pragmatic because the cohort covered three decades. Developmental, cognitive, behavioural and educational information was collected according to age, clinical indication, available instruments, language, school context and routine practice at the time. Formal psychometric or neuropsychological testing was available for some children, but instruments, editions and assessment conditions varied across birth epochs. Cognitive outcome was therefore not analysed as a harmonised continuous IQ or developmental quotient endpoint.

    - **Clinically classified school-age cognitive sequelae** were assigned from the available longitudinal follow-up record, usually around the preschool-to-school transition or the 7-year assessment window. Information sources could include neonatal follow-up, paediatric neurology review, developmental assessment, hospital-based psychometric or neuropsychological testing when available, educational psychology reports, school-based assessments, therapy documentation, school-support records and descriptions of educational functioning. For the main analyses, this outcome was dichotomised as absent versus any clinically classified school-age cognitive sequelae. When enough information was available, cognitive sequelae were classified clinically as absent, mild, moderate or severe. Absence of cognitive sequelae indicated cognitive and educational functioning within the expected range, broadly corresponding to IQ >84 when formal testing was available. Mild sequelae indicated borderline or low-average functioning, broadly corresponding to IQ 71–84, and included mild learning difficulties, minor curricular adaptation or limited school-support needs. Moderate sequelae indicated marked cognitive impairment or intellectual disability, broadly corresponding to IQ 55–70, and

included substantial educational support, significant curricular adaptation or special education. Severe sequelae indicated severe cognitive impairment, broadly corresponding to IQ <55, and included severe intellectual disability, very limited functional communication or severe global developmental disability. These IQ ranges were used as approximate interpretative anchors only and were not treated as uniformly measured numerical endpoints.

- **Sustained developmental/educational support** was analysed as a pragmatic functional outcome intended to capture persistent developmental or educational need beyond isolated early referral. It included maintained therapeutic, developmental or educational support initiated before 2 years, continuing through the preschool period, or first documented at primary-school age. Transient early support only was not classified as sustained support. The type of source used to classify school-age cognitive sequelae and sustained developmental/educational support, such as formal psychometric testing versus broader clinical or educational documentation, was not recorded as a standardised patient-level variable across the full 30-year cohort. Source-stratified analyses were therefore not performed. Analyses involving school-age cognitive sequelae and sustained support should be interpreted as pragmatic clinical follow-up analyses rather than harmonised psychometric endpoint analyses. Follow-up availability and missingness patterns are reported in Supplementary Tables S3 and S4.

Method S8. Supplementary statistical analyses

Continuous variables were summarised as median and interquartile range. Categorical variables were summarised as n/N (%). Descriptive comparisons across phenotype groups used the Mann–Whitney U test, Kruskal–Wallis test, chi-square test or Fisher exact test, as appropriate. P values in descriptive tables were interpreted as indicators of group differences, not as confirmatory hypothesis tests.

The primary adjusted clinical models were fitted within the isolated-IVH analysis framework. The descriptive comparator and model reference group was infants without IVH and without visible APL on serial CUS. Isolated IVH grade 1 and grade 2 were grouped as isolated IVH1–2 in adjusted models because of sparse events, whereas isolated IVH3 was analysed separately. Model A included isolated IVH grade group and was adjusted for gestational age, sex and birth epoch. Model B additionally included severe VM to assess whether ventricular trajectory refined developmental risk stratification beyond maximum IVH grade; this was not interpreted as a formal mediation analysis.

Separate multivariable logistic regression models were fitted for CP, clinically classified school-age cognitive sequelae and sustained developmental/educational support. Results are presented as adjusted odds ratios with 95% confidence intervals. Analyses were performed on an available-case basis without imputation.

Secondary imaging phenotype models assessed whether maximum IVH grade was associated with visible APL, severe VM and hydrocephalus/PHH after adjustment for gestational age, sex and birth epoch. Secondary full CUS phenotype models compared the six mutually exclusive CUS phenotype groups for clinical outcomes, using infants without IVH and without visible APL as the reference group.

Sensitivity and exploratory analyses included models using moderate-to-severe VM, defined as VM≥2, instead of severe VM; exploratory models including hydrocephalus/PHH; analyses of sustained developmental/educational support among children without CP and/or clinically classified school-age cognitive sequelae; Firth penalised-likelihood logistic regression for sparse CP models; and birth-weight-adjusted models replacing gestational age with birth weight.

Firth penalised-likelihood logistic regression was used as a sensitivity analysis for sparse-event CP models involving isolated IVH3 and severe VM. These models used the same covariate set as the primary adjusted models: gestational age, sex and birth epoch.

Given the descriptive and exploratory nature of several subgroup analyses, no adjustment for multiple comparisons was applied. Interpretation emphasised effect estimates, confidence intervals, absolute risks and consistency across analyses rather than statistical significance alone. Analyses were designed to describe associations and refine developmental risk stratification, not to develop or validate a prediction model.

Main analyses were performed using Python version 3.13.5 with pandas version 2.2.3 and statsmodels version 0.14.6.

Supplementary Tables

Supplementary Table S1. Cranial ultrasound availability, cohort assembly and imaging completeness in the study cohort.

Panel A. CUS availability by birth epoch.

Group	Source cohort	≥1 CUS	Complete CUS1	Complete CUS7	Complete CUS28	Complete discharge/TEA CUS	≥2 CUS windows with IVH grade	≥2 complete CUS examinations	All four complete CUS examinations
Overall	3081	2873/3081 (93.2%)	2841/3081 (92.2%)	2719/3081 (88.3%)	2588/3081 (84.0%)	2554/3081 (82.9%)	2773/3081 (90.0%)	2756/3081 (89.5%)	2452/3081 (79.6%)
1991–2000	856	762/856 (89.0%)	752/856 (87.9%)	713/856 (83.3%)	683/856 (79.8%)	674/856 (78.7%)	717/856 (83.8%)	717/856 (83.8%)	655/856 (76.5%)
2001–2010	933	876/933 (93.9%)	867/933 (92.9%)	825/933 (88.4%)	769/933 (82.4%)	754/933 (80.8%)	848/933 (90.9%)	832/933 (89.2%)	726/933 (77.8%)
2011–2020	1292	1235/1292 (95.6%)	1222/1292 (94.6%)	1181/1292 (91.4%)	1136/1292 (87.9%)	1126/1292 (87.2%)	1208/1292 (93.5%)	1207/1292 (93.4%)	1071/1292 (82.9%)

Legend: Values are n/N (%), unless otherwise stated. Complete CUS windows required IVH grade, ventriculomegaly grade and parenchymal lesion pattern to be available.

Panel B. Reasons for exclusion from the main serial CUS cohort

Category	n/N (%)	Reason/status
No cranial ultrasound examination	208/3081 (6.8%)	Excluded from ultrasound cohort
Died before NICU admission	113/208 (54.3%)	Reason for no CUS
Died in NICU before first CUS	90/208 (43.3%)	Reason for no CUS
Transferred to another centre before CUS	5/208 (2.4%)	Reason for no CUS
Only one complete three-domain CUS window among infants with >=1 CUS	117/2873 (4.1%)	Excluded from main serial CUS analysis
**Total excluded from main serial CUS cohort**	**325/3081 (10.5%)**	**No CUS or insufficient serial CUS data**

Legend: Values are n/N (%).

Panel C. CUS availability by maximum IVH grade within the main serial CUS cohort

Group	N	Complete CUS1	Complete CUS7	Complete CUS28	Complete discharge/TEA CUS	All four complete CUS examinations
No IVH	2063	2041/2063 (98.9%)	2036/2063 (98.7%)	1967/2063 (95.3%)	1962/2063 (95.1%)	1881/2063 (91.2%)
Grade 1 IVH	354	350/354 (98.9%)	350/354 (98.9%)	336/354 (94.9%)	321/354 (90.7%)	310/354 (87.6%)
Grade 2 IVH	187	186/187 (99.5%)	184/187 (98.4%)	163/187 (87.2%)	158/187 (84.5%)	155/187 (82.9%)
Grade 3 IVH	152	148/152 (97.4%)	149/152 (98.0%)	122/152 (80.3%)	112/152 (73.7%)	106/152 (69.7%)

Legend: Values are n/N (%). The denominator is the main serial CUS cohort (n=2756). Complete CUS windows required IVH grade, ventriculomegaly grade and parenchymal lesion pattern to be available.

Panel D. CUS availability by CUS phenotype within the main serial CUS cohort

Group	N	Complete CUS1	Complete CUS7	Complete CUS28	Complete discharge/TEA CUS	All four complete CUS examinations
No IVH/no visible APL	1617	1598/1617 (98.8%)	1597/1617 (98.8%)	1544/1617 (95.5%)	1550/1617 (95.9%)	1486/1617 (91.9%)
No IVH + visible APL	446	443/446 (99.3%)	439/446 (98.4%)	423/446 (94.8%)	412/446 (92.4%)	395/446 (88.6%)
Isolated IVH1	259	255/259 (98.5%)	257/259 (99.2%)	246/259 (95.0%)	237/259 (91.5%)	228/259 (88.0%)
Isolated IVH2	94	93/94 (98.9%)	92/94 (97.9%)	81/94 (86.2%)	79/94 (84.0%)	77/94 (81.9%)
Isolated IVH3	60	58/60 (96.7%)	58/60 (96.7%)	51/60 (85.0%)	50/60 (83.3%)	46/60 (76.7%)
IVH + visible APL	280	278/280 (99.3%)	276/280 (98.6%)	243/280 (86.8%)	225/280 (80.4%)	220/280 (78.6%)

Legend: Values are n/N (%). The denominator is the main serial CUS cohort (n=2756). APL denotes associated parenchymal lesion visible on serial CUS. Complete CUS windows required IVH grade, ventriculomegaly grade and parenchymal lesion pattern to be available.

Supplementary Table S2. Baseline characteristics of infants included versus excluded from the main serial CUS analysis

Group	N	Gestational age, weeks	Birth weight, g	Male sex	Multiple gestation	5-min Apgar score	Death before discharge
Included in main serial CUS cohort	2756	29 (27, 31)	1150 (898, 1361)	1435/2756 (52.1%)	854/2751 (31.0%)	9 (7, 9)	196/2756 (7.1%)
Excluded from main serial CUS cohort	325	25 (23, 28)	660 (560, 940)	192/325 (59.1%)	79/308 (25.6%)	4 (1, 8)	316/325 (97.2%)

Legend: Values are median (interquartile range) or n/N (%). The main serial CUS cohort included infants with at least two complete CUS examinations. CUS, cranial ultrasound.

Supplementary Table S3. Developmental follow-up availability by CUS phenotype

CUS phenotype group	Ultrasound N	CP available	School-age cognitive sequelae available	Sustained support available
No IVH, no visible parenchymal lesion	1617	1331/1617 (82.3%)	968/1617 (59.9%)	1277/1617 (79.0%)
No IVH with visible APL	446	362/446 (81.2%)	217/446 (48.7%)	349/446 (78.3%)
Isolated IVH1	259	206/259 (79.5%)	147/259 (56.8%)	192/259 (74.1%)
Isolated IVH2	94	71/94 (75.5%)	44/94 (46.8%)	68/94 (72.3%)
Isolated IVH3	60	40/60 (66.7%)	37/60 (61.7%)	40/60 (66.7%)
IVH with visible APL	280	194/280 (69.3%)	140/280 (50.0%)	182/280 (65.0%)

Legend: Outcome availability by CUS phenotype. School-age cognitive sequelae refers to clinically classified school-age cognitive sequelae; sustained support refers to sustained developmental/educational support. APL, associated parenchymal lesion visible on serial CUS.

Supplementary Table S4. Missingness patterns for developmental follow-up outcomes

Panel A. Missingness pattern for cerebral palsy follow-up

Characteristic	CP follow-up available	CP follow-up missing	p value
Gestational age, weeks, median (IQR)	29 (28, 31)	29 (27, 31)	0.006
Birth weight, g, median (IQR)	1180 (940, 1376)	1152 (850, 1400)	0.256
Male sex	846/1648 (51.3%)	200/382 (52.4%)	0.733
Severe VM	48/1648 (2.9%)	8/382 (2.1%)	0.488
Hydrocephalus/PHH	15/1648 (0.9%)	4/382 (1.0%)	0.769
Birth epoch: 1991-2000	458/1648 (27.8%)	88/382 (23.0%)	0.118
Birth epoch: 2001-2010	558/1648 (33.9%)	130/382 (34.0%)	
Birth epoch: 2011-2020	632/1648 (38.3%)	164/382 (42.9%)	
No IVH/no visible APL	1331/1648 (80.8%)	286/382 (74.9%)	0.003
Isolated IVH1–2	277/1648 (16.8%)	76/382 (19.9%)	
Isolated IVH3	40/1648 (2.4%)	20/382 (5.2%)	

Legend: Restricted to the isolated-IVH analysis cohort.

Panel B. Missingness pattern for clinically classified school-age cognitive sequelae

Characteristic	Cognitive follow-up available	Cognitive follow-up missing	p value
Gestational age, weeks, median (IQR)	29 (27, 31)	29 (27, 31)	0.139
Birth weight, g, median (IQR)	1160 (930, 1360)	1180 (912, 1400)	0.265
Male sex	602/1196 (50.3%)	444/834 (53.2%)	0.206
Severe VM	38/1196 (3.2%)	18/834 (2.2%)	0.215
Hydrocephalus/PHH	10/1196 (0.8%)	9/834 (1.1%)	0.642
Birth epoch: 1991-2000	403/1196 (33.7%)	143/834 (17.1%)	<0.001
Birth epoch: 2001-2010	483/1196 (40.4%)	205/834 (24.6%)	
Birth epoch: 2011-2020	310/1196 (25.9%)	486/834 (58.3%)	
No IVH/no visible APL	968/1196 (80.9%)	649/834 (77.8%)	0.125
Isolated IVH1–2	191/1196 (16.0%)	162/834 (19.4%)	
Isolated IVH3	37/1196 (3.1%)	23/834 (2.8%)	

Legend: Children born in the most recent epoch were over-represented among missing school-age cognitive outcome data.

Panel C. Missingness pattern for sustained developmental/educational support

Characteristic	Support data available	Support data missing	p value
Gestational age, weeks, median (IQR)	30 (28, 31)	29 (27, 31)	0.015
Birth weight, g, median (IQR)	1180 (930, 1380)	1170 (900, 1380)	0.576
Male sex	805/1577 (51.0%)	241/453 (53.2%)	0.424
Severe VM	43/1577 (2.7%)	13/453 (2.9%)	0.871
Hydrocephalus/PHH	13/1577 (0.8%)	6/453 (1.3%)	0.403
Birth epoch: 1991-2000	416/1577 (26.4%)	130/453 (28.7%)	0.141
Birth epoch: 2001-2010	552/1577 (35.0%)	136/453 (30.0%)	
Birth epoch: 2011-2020	609/1577 (38.6%)	187/453 (41.3%)	
No IVH/no visible APL	1277/1577 (81.0%)	340/453 (75.1%)	0.011
Isolated IVH1–2	260/1577 (16.5%)	93/453 (20.5%)	
Isolated IVH3	40/1577 (2.5%)	20/453 (4.4%)	

Legend: Restricted to the isolated-IVH analysis cohort.

Supplementary Table S5. Maximum IVH grade by gestational age and birth epoch

Group	N	No IVH	Grade 1 IVH	Grade 2 IVH	Grade 3 IVH
<26 weeks	324	172/324 (53.1%)	40/324 (12.3%)	70/324 (21.6%)	42/324 (13.0%)
26–27 weeks	495	308/495 (62.2%)	83/495 (16.8%)	55/495 (11.1%)	49/495 (9.9%)
28–29 weeks	687	521/687 (75.8%)	83/687 (12.1%)	38/687 (5.5%)	45/687 (6.6%)
≥30 weeks	1250	1062/1250 (85.0%)	148/1250 (11.8%)	24/1250 (1.9%)	16/1250 (1.3%)
1991–2000	717	551/717 (76.8%)	105/717 (14.6%)	19/717 (2.6%)	42/717 (5.9%)
2001–2010	832	615/832 (73.9%)	102/832 (12.3%)	53/832 (6.4%)	62/832 (7.5%)
2011–2020	1207	897/1207 (74.3%)	147/1207 (12.2%)	115/1207 (9.5%)	48/1207 (4.0%)

Legend: Values are n/N (%).

Supplementary Table S6. Temporal pattern of grade 2 and grade 3 IVH

Maximum IVH grade	Cohort	N	Direct maximum grade at first IVH detection	Progression from lower grade	Maximum grade first reached after CUS7	Severe VM	Hydrocephalus/PHH
Grade 2 IVH	Full cohort	187	155/187 (82.9%)	32/187 (17.1%)	19/187 (10.2%)	9/187 (4.8%)	4/187 (2.1%)
Grade 2 IVH	Isolated IVH	94	74/94 (78.7%)	20/94 (21.3%)	10/94 (10.6%)	4/94 (4.3%)	1/94 (1.1%)
Grade 3 IVH	Full cohort	152	72/152 (47.4%)	80/152 (52.6%)	38/152 (25.0%)	62/152 (40.8%)	33/152 (21.7%)
Grade 3 IVH	Isolated IVH	60	23/60 (38.3%)	37/60 (61.7%)	20/60 (33.3%)	22/60 (36.7%)	9/60 (15.0%)

Legend: Isolated IVH was defined as IVH without associated parenchymal lesion visible on serial CUS. VM, ventriculomegaly.

Supplementary Table S7. Timing of maximum IVH grade

Maximum IVH grade	Cohort	N	CUS1	CUS7	CUS28	Discharge
Grade 2 IVH	Full cohort	187	117/187 (62.6%)	51/187 (27.3%)	17/187 (9.1%)	2/187 (1.1%)
Grade 2 IVH	Isolated IVH	94	59/94 (62.8%)	25/94 (26.6%)	8/94 (8.5%)	2/94 (2.1%)
Grade 3 IVH	Full cohort	152	39/152 (25.7%)	75/152 (49.3%)	36/152 (23.7%)	2/152 (1.3%)
Grade 3 IVH	Isolated IVH	60	11/60 (18.3%)	29/60 (48.3%)	19/60 (31.7%)	1/60 (1.7%)

Legend: Values indicate the scan window in which the maximum IVH grade was first reached.

Supplementary Table S8. Adjusted associations between maximum IVH grade and imaging phenotype components

Imaging phenotype component	Events/N	Grade 1 IVH aOR (95% CI)	Grade 1 IVH p value	Grade 2 IVH aOR (95% CI)	Grade 2 IVH p value	Grade 3 IVH aOR (95% CI)	Grade 3 IVH p value	p for trend
Visible APL	726/2756 (26.3%)	1.26 (0.97–1.65)	0.082	2.69 (1.94–3.73)	<0.001	5.42 (3.76–7.81)	<0.001	<0.001
Severe VM (maximum VM grade 3)	115/2756 (4.2%)	0.91 (0.38–2.18)	0.833	2.26 (1.03–4.95)	0.041	34.15 (20.34–57.34)	<0.001	<0.001
Hydrocephalus/PHH	48/2756 (1.7%)	0.59 (0.08–4.66)	0.62	4.66 (1.36–15.96)	0.014	64.50 (27.76–149.90)	<0.001	<0.001

Legend: Reference group: infants without IVH. Standard logistic regression adjusted for gestational age, sex and birth epoch. APL denotes associated parenchymal lesion visible on serial CUS.

Supplementary Table S9. Associated parenchymal lesions visible on serial CUS according to IVH and birth epoch

Panel A. Associated parenchymal lesion visible on serial CUS by birth epoch and maximum IVH grade

Birth epoch	Maximum IVH grade	N	Associated parenchymal lesion visible on CUS	No associated parenchymal lesion visible on CUS
1991–2000	No IVH	551	110/551 (20.0%)	441/551 (80.0%)
1991–2000	Grade 1 IVH	105	29/105 (27.6%)	76/105 (72.4%)
1991–2000	Grade 2 IVH	19	7/19 (36.8%)	12/19 (63.2%)
1991–2000	Grade 3 IVH	42	25/42 (59.5%)	17/42 (40.5%)
2001–2010	No IVH	615	70/615 (11.4%)	545/615 (88.6%)
2001–2010	Grade 1 IVH	102	21/102 (20.6%)	81/102 (79.4%)
2001–2010	Grade 2 IVH	53	18/53 (34.0%)	35/53 (66.0%)
2001–2010	Grade 3 IVH	62	35/62 (56.5%)	27/62 (43.5%)
2011–2020	No IVH	897	266/897 (29.7%)	631/897 (70.3%)
2011–2020	Grade 1 IVH	147	45/147 (30.6%)	102/147 (69.4%)
2011–2020	Grade 2 IVH	115	68/115 (59.1%)	47/115 (40.9%)
2011–2020	Grade 3 IVH	48	32/48 (66.7%)	16/48 (33.3%)

Legend: This table addresses possible epoch-related changes in detection/reporting of visible parenchymal lesions.

Panel B. Composition of associated parenchymal lesion visible on serial CUS by IVH status and maximum IVH grade

Visible APL category	Overall visible APL N=726	No IVH with visible APL N=446	IVH1 with visible APL N=95	IVH2 with visible APL N=93	IVH3 with visible APL N=92	Any IVH with visible APL N=280
PVHI	100/726 (13.8%)	21/446 (4.7%)	7/95 (7.4%)	26/93 (28.0%)	46/92 (50.0%)	79/280 (28.2%)
Cystic white-matter injury / cPVL	74/726 (10.2%)	41/446 (9.2%)	9/95 (9.5%)	8/93 (8.6%)	16/92 (17.4%)	33/280 (11.8%)
Transient bilateral periventricular echodensities resolving on follow-up	373/726 (51.4%)	274/446 (61.4%)	53/95 (55.8%)	34/93 (36.6%)	12/92 (13.0%)	99/280 (35.4%)
Persistent or unresolved non-cystic periventricular echodensities	126/726 (17.4%)	80/446 (17.9%)	22/95 (23.2%)	15/93 (16.1%)	9/92 (9.8%)	46/280 (16.4%)
Arterial infarction	7/726 (1.0%)	4/446 (0.9%)	0/95 (0.0%)	1/93 (1.1%)	2/92 (2.2%)	3/280 (1.1%)
Parenchymal haemorrhage, non-PVHI	12/726 (1.7%)	4/446 (0.9%)	1/95 (1.1%)	4/93 (4.3%)	3/92 (3.3%)	8/280 (2.9%)
Deep grey matter lesion	21/726 (2.9%)	15/446 (3.4%)	2/95 (2.1%)	4/93 (4.3%)	0/92 (0.0%)	6/280 (2.1%)
Multicystic encephalomalacia	10/726 (1.4%)	6/446 (1.3%)	1/95 (1.1%)	1/93 (1.1%)	2/92 (2.2%)	4/280 (1.4%)
Mixed focal lesion patterns / other major focal lesions	3/726 (0.4%)	1/446 (0.2%)	0/95 (0.0%)	0/93 (0.0%)	2/92 (2.2%)	2/280 (0.7%)
**Total**	**726/726 (100.0%)**	**446/446 (100.0%)**	**95/95 (100.0%)**	**93/93 (100.0%)**	**92/92 (100.0%)**	**280/280 (100.0%)**

Legend: Values are n/N (%). Percentages are column percentages. APL denotes associated parenchymal lesion visible on serial CUS. Categories were mutually exclusive and derived from registry-recorded serial CUS findings.

Supplementary Table S10. Adjusted associations of full CUS phenotype with clinical outcomes

Outcome	Events/N	CUS phenotype	aOR (95% CI)	p value
Cerebral palsy	142/2204 (6.4%)	No IVH with visible APL	12.81 (7.37-22.26)	<0.001
Cerebral palsy	142/2204 (6.4%)	Isolated IVH1	1.97 (0.78-5.02)	0.154
Cerebral palsy	142/2204 (6.4%)	Isolated IVH2	1.79 (0.40-7.98)	0.447
Cerebral palsy	142/2204 (6.4%)	Isolated IVH3	22.15 (9.39-52.26)	<0.001
Cerebral palsy	142/2204 (6.4%)	IVH with visible APL	25.64 (14.39-45.71)	<0.001
School-age cognitive sequelae	165/1553 (10.6%)	No IVH with visible APL	2.20 (1.40-3.44)	<0.001
School-age cognitive sequelae	165/1553 (10.6%)	Isolated IVH1	1.14 (0.62-2.08)	0.672
School-age cognitive sequelae	165/1553 (10.6%)	Isolated IVH2	1.39 (0.55-3.48)	0.487
School-age cognitive sequelae	165/1553 (10.6%)	Isolated IVH3	2.84 (1.25-6.44)	0.012
School-age cognitive sequelae	165/1553 (10.6%)	IVH with visible APL	2.44 (1.47-4.05)	<0.001
Sustained developmental/educational support	479/2108 (22.7%)	No IVH with visible APL	1.50 (1.12-2.00)	0.007
Sustained developmental/educational support	479/2108 (22.7%)	Isolated IVH1	0.97 (0.66-1.44)	0.891
Sustained developmental/educational support	479/2108 (22.7%)	Isolated IVH2	1.64 (0.95-2.84)	0.076
Sustained developmental/educational support	479/2108 (22.7%)	Isolated IVH3	2.93 (1.52-5.66)	0.001
Sustained developmental/educational support	479/2108 (22.7%)	IVH with visible APL	3.04 (2.16-4.27)	<0.001

Legend: Reference group: no IVH and no associated parenchymal lesion visible on serial CUS. Models were adjusted for gestational age, sex and birth epoch. This was a secondary contextual analysis used to assess the contribution of the full serial CUS phenotype.

Supplementary Table S11. Cerebral palsy type and severity according to CUS phenotype

Characteristic	No IVH/no visible APL	No IVH + visible APL	Isolated IVH1	Isolated IVH2	Isolated IVH3	IVH + visible APL
Phenotype group N	1617	446	259	94	60	280
CP assessed	1331/1617 (82.3%)	362/446 (81.2%)	206/259 (79.5%)	71/94 (75.5%)	40/60 (66.7%)	194/280 (69.3%)
Any cerebral palsy	19/1331 (1.4%)	52/362 (14.4%)	6/206 (2.9%)	2/71 (2.8%)	11/40 (27.5%)	52/194 (26.8%)
CP type among children with CP						
Monoplegia	1/19 (5.3%)	0/52 (0.0%)	0/6 (0.0%)	0/2 (0.0%)	0/11 (0.0%)	8/52 (15.4%)
Hemiplegia	5/19 (26.3%)	14/52 (26.9%)	1/6 (16.7%)	1/2 (50.0%)	0/11 (0.0%)	22/52 (42.3%)
Spastic diplegia	10/19 (52.6%)	21/52 (40.4%)	1/6 (16.7%)	1/2 (50.0%)	10/11 (90.9%)	12/52 (23.1%)
Triparesis	0/19 (0.0%)	1/52 (1.9%)	2/6 (33.3%)	0/2 (0.0%)	0/11 (0.0%)	1/52 (1.9%)
Quadriplegia	2/19 (10.5%)	14/52 (26.9%)	2/6 (33.3%)	0/2 (0.0%)	0/11 (0.0%)	7/52 (13.5%)
Other spastic-dystonic/ataxic	1/19 (5.3%)	2/52 (3.8%)	0/6 (0.0%)	0/2 (0.0%)	1/11 (9.1%)	2/52 (3.8%)
GMFCS level among children with CP						
GMFCS level I	9/19 (47.4%)	16/52 (30.8%)	3/6 (50.0%)	1/2 (50.0%)	6/11 (54.5%)	25/52 (48.1%)
GMFCS level II	5/19 (26.3%)	11/52 (21.2%)	0/6 (0.0%)	1/2 (50.0%)	3/11 (27.3%)	14/52 (26.9%)
GMFCS level III	2/19 (10.5%)	16/52 (30.8%)	1/6 (16.7%)	0/2 (0.0%)	1/11 (9.1%)	5/52 (9.6%)
GMFCS level IV	1/19 (5.3%)	4/52 (7.7%)	2/6 (33.3%)	0/2 (0.0%)	1/11 (9.1%)	4/52 (7.7%)
GMFCS level V	2/19 (10.5%)	5/52 (9.6%)	0/6 (0.0%)	0/2 (0.0%)	0/11 (0.0%)	4/52 (7.7%)

Legend: Values are n/N (%) unless otherwise stated. CP assessed denotes children with available cerebral palsy status. CP type and GMFCS level were summarised among children with CP. APL denotes associated parenchymal lesion visible on serial CUS. CP, cerebral palsy; CUS, cranial ultrasound; GMFCS, Gross Motor Function Classification System.

Supplementary Table S12. Cerebral palsy according to refined visible APL category

Refined CUS phenotype	Phenotype N	CP assessed	Any CP	Hemiplegia among CP	Spastic diplegia among CP	Triparesis/quadriplegia among CP	GMFCS level I-II among CP	GMFCS level III-V among CP
No IVH/no visible APL	1617	1331/1617 (82.3%)	19/1331 (1.4%)	5/19 (26.3%)	10/19 (52.6%)	2/19 (10.5%)	14/19 (73.7%)	5/19 (26.3%)
Isolated IVH1-2	353	277/353 (78.5%)	8/277 (2.9%)	2/8 (25.0%)	2/8 (25.0%)	4/8 (50.0%)	5/8 (62.5%)	3/8 (37.5%)
Isolated IVH3	60	40/60 (66.7%)	11/40 (27.5%)	0/11 (0.0%)	10/11 (90.9%)	0/11 (0.0%)	9/11 (81.8%)	2/11 (18.2%)
No IVH + transient bilateral periventricular echodensities resolving on follow-up	274	239/274 (87.2%)	12/239 (5.0%)	3/12 (25.0%)	6/12 (50.0%)	2/12 (16.7%)	10/12 (83.3%)	2/12 (16.7%)
IVH + transient bilateral periventricular echodensities resolving on follow-up	99	84/99 (84.8%)	7/84 (8.3%)	2/7 (28.6%)	3/7 (42.9%)	1/7 (14.3%)	6/7 (85.7%)	1/7 (14.3%)
No IVH + persistent or unresolved non-cystic periventricular echodensities	80	51/80 (63.7%)	0/51 (0.0%)	—	—	—	—	—
IVH + persistent or unresolved non-cystic periventricular echodensities	46	25/46 (54.3%)	3/25 (12.0%)	1/3 (33.3%)	0/3 (0.0%)	1/3 (33.3%)	1/3 (33.3%)	2/3 (66.7%)
No IVH + major visible parenchymal lesion	92	72/92 (78.3%)	40/72 (55.6%)	11/40 (27.5%)	15/40 (37.5%)	13/40 (32.5%)	17/40 (42.5%)	23/40 (57.5%)
IVH + major visible parenchymal lesion	135	85/135 (63.0%)	42/85 (49.4%)	19/42 (45.2%)	9/42 (21.4%)	6/42 (14.3%)	32/42 (76.2%)	10/42 (23.8%)
of which: IVH3 + transient bilateral periventricular echodensities resolving on follow-up	12	11/12 (91.7%)	1/11 (9.1%)	0/1 (0.0%)	1/1 (100.0%)	0/1 (0.0%)	1/1 (100.0%)	0/1 (0.0%)
of which: IVH3 + persistent or unresolved non-cystic periventricular echodensities	9	4/9 (44.4%)	2/4 (50.0%)	0/2 (0.0%)	0/2 (0.0%)	1/2 (50.0%)	0/2 (0.0%)	2/2 (100.0%)
of which: IVH3 + major visible parenchymal lesion	71	35/71 (49.3%)	20/35 (57.1%)	9/20 (45.0%)	4/20 (20.0%)	3/20 (15.0%)	15/20 (75.0%)	5/20 (25.0%)

Legend: Values are n/N (%). APL denotes associated parenchymal lesion visible on serial CUS. This refined analysis separated transient bilateral periventricular echodensities resolving on follow-up from persistent or unresolved non-cystic periventricular echodensities and from major visible parenchymal lesion. These analyses were secondary and descriptive. They were intended to contextualise heterogeneity within the broad visible APL composite and should not be interpreted as validated lesion-specific prognostic estimates.

Supplementary Table S13. Sensitivity models using moderate-to-severe VM (VM≥2) instead of severe VM

Outcome	Model	Events/N	Isolated IVH1-2 aOR (95% CI)	p	Isolated IVH3 aOR (95% CI)	p	VM≥2 aOR (95% CI)	p
Cerebral palsy	Model A: IVH grade group	38/1648 (2.3%)	2.12 (0.90-4.99)	0.085	28.55 (11.31-72.11)	<0.001	-	-
Cerebral palsy	Model B: IVH grade group + VM≥2	38/1648 (2.3%)	2.05 (0.87-4.85)	0.102	16.12 (5.80-44.81)	<0.001	3.25 (1.27-8.31)	0.014
School-age cognitive sequelae	Model A: IVH grade group	105/1196 (8.8%)	1.18 (0.69-2.01)	0.542	2.67 (1.17-6.12)	0.020	-	-
School-age cognitive sequelae	Model B: IVH grade group + VM≥2	105/1196 (8.8%)	1.17 (0.68-1.99)	0.570	2.09 (0.84-5.24)	0.114	1.53 (0.79-2.95)	0.210
Sustained developmental/educational support	Model A: IVH grade group	314/1577 (19.9%)	1.12 (0.80-1.56)	0.518	2.88 (1.48-5.58)	0.002	-	-
Sustained developmental/educational support	Model B: IVH grade group + VM≥2	314/1577 (19.9%)	1.10 (0.79-1.54)	0.582	2.15 (1.05-4.39)	0.036	1.65 (1.07-2.55)	0.024

Legend: Reference group: infants without IVH and without visible APL. Models were adjusted for gestational age, sex and birth epoch. Model A included isolated IVH grade group. Model B additionally included moderate-to-severe VM. Moderate-to-severe VM was defined as maximum VM grade ≥2 across serial CUS.

Supplementary Table S14. Exploratory models with hydrocephalus/PHH

Outcome	Model	Events/N	Isolated IVH1-2 aOR (95% CI)	p	Isolated IVH3 aOR (95% CI)	p	Hydrocephalus/PHH aOR (95% CI)	p
Cerebral palsy	Model B: IVH grade group + hydrocephalus/PHH	38/1648 (2.3%)	2.19 (0.92-5.21)	0.077	13.87 (4.80-40.10)	<0.001	37.53 (9.57-147.24)	<0.001
School-age cognitive sequelae	Model B: IVH grade group + hydrocephalus/PHH	105/1196 (8.8%)	1.16 (0.68-1.99)	0.580	1.55 (0.58-4.09)	0.381	20.57 (4.78-88.55)	<0.001
Sustained developmental/educational support	Model B: IVH grade group + hydrocephalus/PHH	314/1577 (19.9%)	1.12 (0.80-1.56)	0.515	2.35 (1.17-4.72)	0.017	4.63 (1.36-15.78)	0.014

Legend: Reference group: infants without IVH and without visible APL. Models were adjusted for gestational age, sex and birth epoch. Hydrocephalus/PHH was analysed as a documented clinicoradiological ventricular complication during neonatal admission. In infants with IVH, hydrocephalus was considered post-haemorrhagic hydrocephalus (PHH).

Supplementary Table S15. Sustained developmental/educational support analyses in the isolated-IVH framework

Panel A. Sustained developmental/educational support among children without cerebral palsy and/or school-age cognitive sequelae

Subgroup	No IVH/no visible APL	Isolated IVH1	Isolated IVH2	Isolated IVH3
Without cerebral palsy	218/1246 (17.5%)	32/185 (17.3%)	21/66 (31.8%)	10/28 (35.7%)
Without school-age cognitive sequelae	137/888 (15.4%)	17/130 (13.1%)	10/38 (26.3%)	10/28 (35.7%)
Without cerebral palsy and without school-age cognitive sequelae	133/882 (15.1%)	16/127 (12.6%)	10/37 (27.0%)	5/21 (23.8%)

Panel B. Exploratory adjusted association between severe VM and sustained developmental/educational support

Subgroup	Severe VM aOR (95% CI)
Without cerebral palsy	2.40 (1.11-5.18)
Without school-age cognitive sequelae	3.94 (1.65-9.41)
Without cerebral palsy and without school-age cognitive sequelae	3.78 (1.50-9.50)

Legend: Values in Panel A are n/N (%). Analyses were restricted to children in the isolated-IVH analysis framework, defined as infants without associated parenchymal lesion visible on serial CUS. Sustained developmental/educational support was analysed as a pragmatic functional outcome. Panel B models were adjusted for gestational age, sex and birth epoch. Severe VM was defined as maximum VM grade 3 across serial CUS.

Supplementary Table S16. Firth penalised-likelihood logistic regression sensitivity analyses for cerebral palsy

Outcome	Model	Events/N	Isolated IVH1-2 penalised aOR (95% CI)	Isolated IVH1-2 p value	Isolated IVH3 penalised aOR (95% CI)	Isolated IVH3 p value	Severe VM penalised aOR (95% CI)	Severe VM p value
Cerebral palsy	Firth Model A: IVH grade group	38/1648 (2.3%)	2.18 (0.97-4.94)	0.061	27.56 (11.13-68.23)	<0.001	-	-
Cerebral palsy	Firth Model B: IVH grade group + severe VM	38/1648 (2.3%)	2.13 (0.94-4.83)	0.071	12.45 (4.39-35.32)	<0.001	6.16 (2.17-17.53)	<0.001

Legend: Firth penalised-likelihood logistic regression was used as a robustness check because cerebral palsy events were sparse in isolated IVH3 and severe VM subgroups. Models included the same adjustment set as the primary logistic models: gestational age, sex and birth epoch. Values are penalised adjusted odds ratios with 95% confidence intervals.

Supplementary Table S17. Birth-weight-adjusted sensitivity analysis replacing gestational age in primary adjusted models

Outcome	Model	Events/N	Isolated IVH1–2 aOR (95% CI)	Isolated IVH1–2 p value	Isolated IVH3 aOR (95% CI)	Isolated IVH3 p value	Severe VM aOR (95% CI)	Severe VM p value
Cerebral palsy	Model A: IVH grade group, adjusted for birth weight, sex and birth epoch	38/1648 (2.3%)	2.08 (0.89–4.83)	0.090	27.29 (11.50–64.75)	<0.001	—	—
Cerebral palsy	Model B: IVH grade group + severe VM, adjusted for birth weight, sex and birth epoch	38/1648 (2.3%)	2.02 (0.86–4.72)	0.105	12.39 (4.44–34.58)	<0.001	6.15 (2.11–17.96)	<0.001
School-age cognitive sequelae	Model A: IVH grade group, adjusted for birth weight, sex and birth epoch	105/1196 (8.8%)	1.19 (0.70–2.02)	0.525	2.71 (1.20–6.13)	0.017	—	—
School-age cognitive sequelae	Model B: IVH grade group + severe VM, adjusted for birth weight, sex and birth epoch	105/1196 (8.8%)	1.16 (0.68–1.98)	0.586	1.90 (0.76–4.76)	0.170	2.33 (0.97–5.61)	0.059
Sustained developmental/educational support	Model A: IVH grade group, adjusted for birth weight, sex and birth epoch	314/1577 (19.9%)	1.13 (0.81–1.58)	0.470	3.03 (1.56–5.88)	0.001	—	—
Sustained developmental/educational support	Model B: IVH grade group + severe VM, adjusted for birth weight, sex and birth epoch	314/1577 (19.9%)	1.11 (0.79–1.56)	0.537	1.90 (0.91–3.96)	0.087	3.23 (1.61–6.46)	<0.001

Legend: Model A included isolated IVH grade group and was adjusted for birth weight, sex and birth epoch. Model B additionally included severe VM. Severe VM was defined as maximum VM grade 3 across serial CUS. Reference group: infants without IVH and without visible APL. APL denotes associated parenchymal lesion visible on serial CUS.

Supplementary Table S18. Ventriculomegaly severity mapping used in the study

Severity grade	Study category	Thalamo-occipital distance (TOD)	Atrial (trigonal) diameter
0	None	<20 mm	<10 mm
1	Mild	20–24.9 mm	10–12 mm
2	Moderate	25–29.9 mm	13–15 mm
3	Severe	≥30 mm	>15 mm

Legend: Values are inner-edge to inner-edge ultrasound measurements. Thalamo-occipital distance was measured on a true parasagittal view through the atrium/occipital horn. Atrial width was measured on a true axial transventricular view at the ventricular atrium. The table shows the practical mapping used to support the registry-based VM severity grade; raw quantitative measurements were not uniformly available for every scan across the three-decade cohort.
